# A Single Sub‐Millimetric Metasurface‐Based Optical Element for Lattice Bessel Beam Excitation Enabling Brain Activity Recordings In Vivo

**DOI:** 10.1002/smll.202409258

**Published:** 2025-02-02

**Authors:** Anna Archetti, Matteo Bruzzone, Giulia Tagliabue, Marco dal Maschio

**Affiliations:** ^1^ Department of Biomedical Sciences University of Padua Padua 35131 Italy; ^2^ École Polytechnique Fédérale de Lausanne (EPFL) Lausanne 1015 Switzerland; ^3^ Padova Neuroscience Center – PNC University of Padua Padua 35131 Italy

**Keywords:** bessel beams, brain activity, flat optics, light‐sheet microscopy, metasurfaces

## Abstract

Bessel beams (BBs) are propagation‐invariant optical fields that retain a narrow central intensity profile over longer propagation lengths than Gaussian beams (GBs). Due to this property, they have been adopted in fluorescence‐based light sheet microscopy (LSM) to obtain 2D longitudinally‐extended light‐sheets. Yet, current approaches for generating BB lattices in LSM focus on regular excitation patterns and involve complex and bulky optics, limiting integration capability and versatility. Here, a flexible method is presented to obtain BB‐arrays with arbitrary geometries by encoding on a single sub‐millimetric surface all the optical transformations required. This method is applied using a single metasurface to encode the generation of a linear array of BBs, avoiding the use of conjugation and focusing optics. With respect to the current strategies, this approach, allowing for the independent design of each beamlet of the array, increases the degrees of freedom while making optimal use of the available light with no rejection, thus facilitating its integration into optical systems. According to this method, we fabricated a metasurface‐based optical element for generating a linear BB‐array of excitation in an LSM configuration and recorded neuronal activity at cellular resolution from the zebrafish larva brain. Thus, the proposed approach greatly extends the BB‐array versatility and the application scenarios.

## Graphical abstract

A method to generate a lattice light sheet (LLS) with a single sub‐millimetric optical element and to overcome some limitations of current LLS microscopes. It reduces alignment and geometrical constraints linked with the presence of multiple bulky components, it avoids major rejection or structuring of the input light otherwise necessary in a single delta‐ring approach, and it decouples the numerical aperture of the LLS focusing lens from the lateral dimension of the LLS and thus the LLS lateral extension from the LLS thickness.

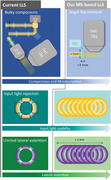

## 1 Introduction

In the last years, Bessel Beams (BBs) and BB arrays have gained in popularity in a number of light‐based applications.^[^ ^1^, ^2^, ^3^, ^4^, ^5^, ^6^ ^]^ With respect to traditional Gaussian beams, BBs present a more elongated point spread function along the propagation direction and a typical cross‐section with a narrower central peak surrounded by lower‐intensity side lobes.^[^ ^7^, ^8^ ^]^ Such an extended longitudinal intensity profile has prompted the adoption of BB from material science analysis to photo‐assisted fabrication methods and optical microscopy. Among all applications of BBs, light sheet microscopy (LSM) has benefited the most. LSM is a fundamental fluorescence imaging technique for structural and functional non‐invasive studies in life science. This method ensures at the same time high signal collection efficiency, reduced out‐of‐plane excitation, and low photodamage.^[^ ^9^ ^]^ In an LSM system, the sample is typically excited with a few micrometers thick sheet of light, and the signal is collected at once from the entire Field of View (FoV) along the direction orthogonal to the illuminated area. Typically, a suitable excitation beam, with a flattened profile in the direction perpendicular to the light propagation direction, is realized by collimating a Gaussian beam onto a cylindrical lens,^[^ ^9^ ^]^ or by using the diffraction of a beam entering the sample glass slide at high angles through an objective^[^ ^10^, ^11^, ^12^, ^13^ ^]^ or a prism.^[^ ^14^ ^]^ In other common solutions, usually referred to as digitally scanned light‐sheet microscopy (DSLM),^[^ ^15^ ^]^ a virtual light sheet is obtained by scanning in the transversal direction a beam across the sample. Along with the traditional use of a single Gaussian beam, more recently, approaches based on an array of Bessel Beams (BBs), such as lattice light sheet (LLS)^[^ ^5^, ^16^, ^17^ ^]^ and universal lattice light sheet,^[^ ^18^ ^]^ have been developed. In the LLS approach, the BBs are distributed along the transversal direction forming a linear regular array, so as to produce a destructive interference of their side lobes while maintaining their narrow intensity profile invariant over several propagation lengths. Such a scheme has rendered BB arrays ideal for light sheet microscopy with improved resolution, excitation profile extension, and reduced phototoxicity.^[^ ^5^, ^16^, ^17^ ^]^

However, the generation of multiple BBs requires specialized optical elements that make the overall optical layout complex and spatially extended. In fact, moving from Gaussian Beam (GB) to BB‐based excitation requires the integration of wavefront modulation components, e.g. an axicon phase plate or an apodization filter (annular mask),^[^ ^8^ ^]^ to render a more elongated excitation profile. Furthermore, the generation of regular lattices of BBs at the sample, like in LLS, is frequently based on beam‐multiplexing elements, like spatial light modulators (SLMs)^[^ ^16^, ^19^ ^]^ or other diffractive optical elements (DOEs).^[^ ^5^ ^]^ Finally, in order to have a proper optical conjugation at the sample by means of the excitation objective, these optical components need to be integrated at different positions along the optical path. On the other side, Meta‐Surface (MS) technology, i.e., nanostructured surfaces that achieve control of light properties, including amplitude, phase, and polarization with sub‐wavelength resolution,^[^ ^20^ ^]^ have benefited many optical systems (e.g., miniaturized lensing systems), but their applications to LSM in a living organism have been rather limited. Moreover, state‐of‐the‐art methods to generate Bessel beam arrays with one single optical element operating in the visible range,^[^ ^21^, ^22^, ^23^ ^]^ typically suffer from some limitations: i) rejection of more than 50% of the input light or limitation of the active area required for LLS generation to less than 50%;^[^ ^22^ ^]^ ii) trade‐off between the active area size, the number of beams and the beam waist;^[^ ^21^, ^22^, ^23^ ^]^ iii) or they are not designed to generate linear arrays.^[^ ^21^, ^23^ ^]^

Here, we overcome the complexity of current bulky LLS microscopy systems, the traditional approaches for the generation of regular BB‐arrays, and the current metasurface‐based BB array approaches. We introduce a single plane optical manipulation method that can generate an array of Bessel beams, not just according to regular geometries, but where potentially each beamlet of the envelope could be independently designed. The method satisfies the required optical conjugations and makes a more efficient use of the available light, at the same time increasing the flexibility. Specifically, combining methods of wavefront engineering and metasurface beam multiplexing capability,^[^ ^24^ ^]^ we developed a design that encodes the transformations required for both the generation of a BB profile, the beam multiplexing, and their conjugation at the sample, so to render an array with multiple and potentially independent BB‐beamlets. Accordingly, we fabricated and characterized a sub‐millimetric metasurface, confirming the generation of the expected BB array patterns. Integrating such MS‐based BB‐excitation in a custom LSM configuration, we demonstrate the recording of neuronal activity from the brain of a zebrafish larva with a spatial resolution sufficient to resolve the individual cells. Thus, our design greatly improves the compactness of LSM systems favoring their widespread deployment.

## 2 Results

### 2.1 Design Principles for Generating a single BB by Wavefront Modulation on a Single Plane

**Figure 1:**
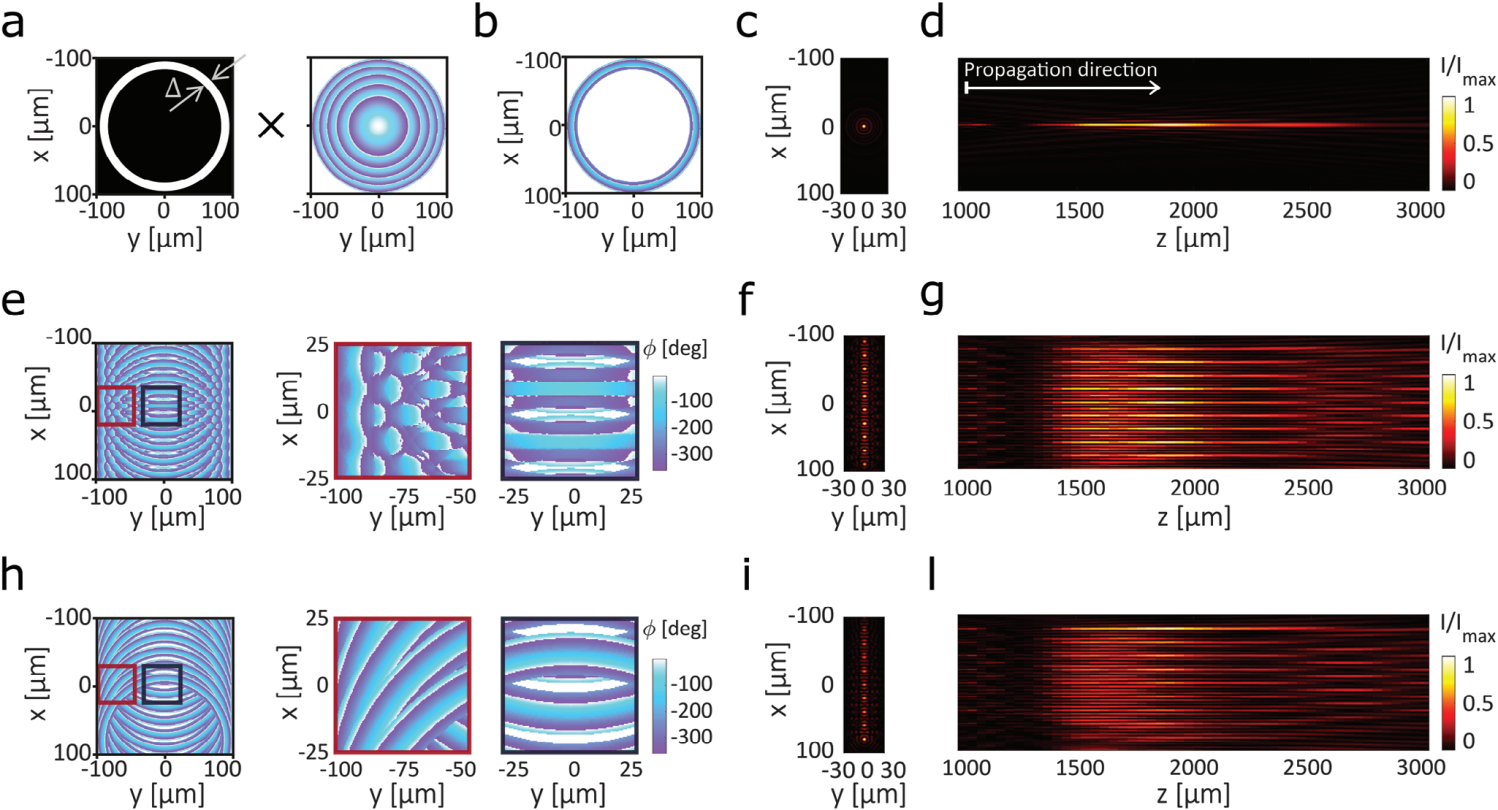
Working principle of the proposed single element Bessel Beam Lattice (BBL) excitation – a) The amplitude annular mask (a‐left) with outer radius *R*  =  100 µ*m*, and Δ  =  17 µ*m* is combined with the phase profile of a convergent lens with focal length *f*  =  2000 µ*m* (a‐right) to produce a BB with a diameter of 6 µm b) Phase profile resulting from the operation described in (a) for the generation of a single BB, black stands for field intensity equal to zero. c) BB intensity profile in the plane transversal to the beam propagation direction; d) BB intensity profile in the beam propagation plane XZ. e) Total phase profile ϕ_***TOT***_ retrieved with Method‐S1 (Supporting Information) for BBL generation (e left). Highlighted regions in e‐left show the phase profile where the rings overlap on the left (e middle) and in the middle (e right). f,g) Simulations of the BB intensity profiles generated with the phase mask ϕ_***j***_ described in e. f, intensity profile of the BB in the XY plane; g) intensity profile of the BB in the XZ plane. h) The BB phase profile ϕ_***TOT***_ generated with Method‐S2 (Supporting Information). Highlighted regions in h left show the phase profile where the rings overlap at the left (h middle) and in the middle (h right). i,l) Simulations of the BB intensity profiles generated from the phase mask shown in h. i) intensity profile of the BB in the XY plane; l) intensity profile of the BB in the XZ plane.

The angular spectrum of a Bessel beam (BB) can be described as a Ring‐Delta function,^[^ ^8^, ^25^, ^26^ ^]^ or as the sum of wavevectors lying on the surface of a single cone. Thus, confining the field intensity of an input Gaussian beam (GB) to a thin ring in the source space results in a BB in the infinite image space or in the corresponding Fourier space of a converging lens/objective.^[^ ^7^, ^8^ ^]^ This method adopts an apodization filter, to select a ring‐shaped portion of the input GB. The apodization filter is then typically conjugated by a 4f optical arrangement to the back focal plane of an objective, to obtain the final BB intensity profile at the sample. Here, we propose a wavefront engineering method combining amplitude and phase modulation to encode on the same optical plane, z_0_, both the apodization filter transforming a GB in a BB and the optical manipulations for its conjugation in the image space. First, we considered an input GB propagating along the *Z*‐direction with a planar wavefront aligned along the *XY*‐plane. Then, we defined an amplitude and phase modulation of its wavefront to obtain, at the plane z_0,_ a field with non‐null intensity distribution within the ring region $R-\Delta \leq \sqrt{x^{2}+y^{2}\;}\leq R$ and zero elsewhere, and with a phase distribution in this support $\phi^{ring}\left(x,y\right)=-\frac{2\pi }{\lambda }\left[\sqrt{x^{2}+y^{2}+f^{2}}-f\right]$. This modulation operates at the same time as an amplitude annular mask and an optical Fourier transformer. The amplitude annular mask depends on two parameters, the external radius *R* of the ring and its thickness Δ, while the Fourier transformation is characterized by the focal length *f* of the corresponding focusing lens (**Figure** **1**a,b). The annulus thickness ∆ allows to tuning of the Gaussian‐over‐Bessel feature balance, while the radius R and focal length *f* allow for controlling the beam longitudinal (*Z*) position along with its longitudinal and transversal Full‐Width‐at‐Half‐Maximum (*FWHM_z_* and *FWHM*_*x*,*y*_ respectively). Indeed, a beam generated by an annulus mask with a finite aperture will have a mix of features from both Gaussian and Bessel beams. The thinner the annulus Δ, the more prominent the Bessel contribution: the central lobe of the beam becomes thinner and the longitudinal dimension of the illumination profile is more extended.

To evaluate the outcome of the developed method, we implemented an iterative wavefront propagation algorithm for calculating the intensity distribution of the propagated beams at the output of the modulation plane z_0_ (see Experimental Section). We thus calculated a wavefront modulation for generating a BB with a *FWHM*_*x*,*y*_ of ≈4 µm, and *FWHM_z_* of 700 µm (design parameters: outside ring radius *R*  =  100 µm, Δ  =  17 µm, focal length *f*  =  2000 µm, wavelength = 488 nm). The analysis of the resulting profiles confirmed that such single plane wavefront modulation pattern can generate BBs close to the design parameters (*FWHM*_*x*,*y*_ ≈4 µm, and *FWHM_z_* ≈700 µm) (Figure 1c,d).

We note that such value of *FWHM*_*x*,*y*_ derives from the design selection and not from an intrinsic constraint of the approach (Note S6, Figures S12–S18, Supporting Information).

Indeed, this approach can be exploited also in applications requiring micrometric or sub‐micrometric light sheet illuminations as those shown in Figures S12 and S15 (Supporting Information).

### 2.2 Generating BB Arrays by WaveFront Modulation and Multiplexing on the Same Plane

For imaging applications, it is common to use BBs in a lattice‐based illumination scheme,^[^ ^5^, ^16^, ^17^ ^]^ with multiple BBs illuminating the sample at the same time in different lateral positions. Optical lattices are periodic interference patterns, organized in 1D or 2D regular arrays of replica beamlets, generated by the coherent superposition of a finite number of plane waves.^[^ ^27^ ^]^ It is known that the period *P* separating the BBs can be optimized to produce a destructive interference between the side secondary rings of adjacent BBs, maximizing the energy directed in the main lobes and minimizing the effective beam diameter.^[^ ^16^ ^]^ Interestingly, we noticed that it is possible to extend our original approach and encode on the same plane a total phase mask ϕ_***TOT***_ capable of generating an array of BBs with the desired geometry in terms of thickness and extension. We considered two multiplexing approaches for obtaining the required phase profile. In the first case, (Method‐1), the total phase profile ϕ_***TOT***_ results from the argument of the sum of the fields *E*_***j***_ each corresponding to the *j‐th* Bessel beam (Figure 1e**–**g): $\phi_{TOT}=arg\left\{\sum_{j\;=1}^{N}E_{j}\right\}\;\mathrm{where}\;\;E_{j}=E_{0j}\;e^{i\phi_{j}}$. Each field *E*_***j***_ is characterized by a phase ϕ_*j*_(*x*, *y*)* * = ϕ^*ring*^ **(*x* − *jP*,  *y*), and an amplitude

(1) $$E_{0j}\left(x,y\right)=\left\{\begin{array}{c} 1,R-\Delta \leq \sqrt{{\left(x-jP\right)}^{2}+y^{2}\;}\leq \;R\\ 0,\;elsewhere \end{array}\right.$$

replica at a *x*‐distance *jP* of the original wavefront modulation described above. In the second approach (Method‐2), the total phase profile ϕ_***TOT***_ for 1D lattice of BBs is generated by overwriting each consecutive field $E_{0j}e^{i\phi_{j}}$ (Figure 1h**–**l; Note S1, Supporting Information). We used this latter approach to design a target phase profile capable of generating a Bessel Beam Lattice (BBL) with a geometry characterized by a thickness (*FWHM_y_*) of ≈6 µm, a lateral extension along the *x*‐axis of ≈200 µm and longitudinal extension (*FWHM_z_*) of 700 µm. We obtained the target phase profile, for the whole lattice, by summing up ten previously synthesized modulation patterns for the single BB, but each laterally shifted in the *x*‐direction by $P∼j\times \frac{\lambda }{NA_{\min}}∼20\mu m$ (Figure 1h–l; for further info on Method‐2‐ see Note S1, Supporting Information). Our beam propagation algorithm confirmed that such single plane wavefront modulation patterns result in a BBL presenting an overall XYZ‐extension of ≈ 200  × 7  × 700 µm^3^ (*FWHM_xyz_*), close to the design parameters. According to our simulations, the developed approach can generate BB arrays with customizable geometries, in particular extending the lattice laterally in the X‐dimension, reducing the beamlets diameter to submicrometric range or changing the beamlets focal positions without affecting the other optical parameters (Note S6, Figures S12–S18, Supporting Information).

Among these two approaches, the first (Method‐1) allows the generation of arrays of Bessel beams with optimal uniformity. Although Method‐2 generates a less homogeneous array, it has been our choice for the demonstration of the approach and of the fabrication process because the presence of a macroscopic pattern repeating itself over the chip surfaces allows one to directly assess the actual performances of each single Bessel beam and to check the fabrication process over different chip area patterned with the same structure (see Note S1, Supporting Information for further info).

It is also worth mentioning that these methods to synthesize BBLs are compatible with the beam dithering methods, i.e., introducing a tilt in the input wavefront, used to generate a continuous sheet of excitation (Note S5, Figures S10 and S11, Supporting Information). Importantly, with respect to current approaches, this flexible approach, along with regular geometry lattices, can generate BB arrays with any arbitrary geometries with the possibility of tuning the parameters of each beamlet independently (Figure S23, Supporting Information).

### 2.3 Generation of BB‐Arrays Using Meta‐Surface Technology

**Figure 2:**
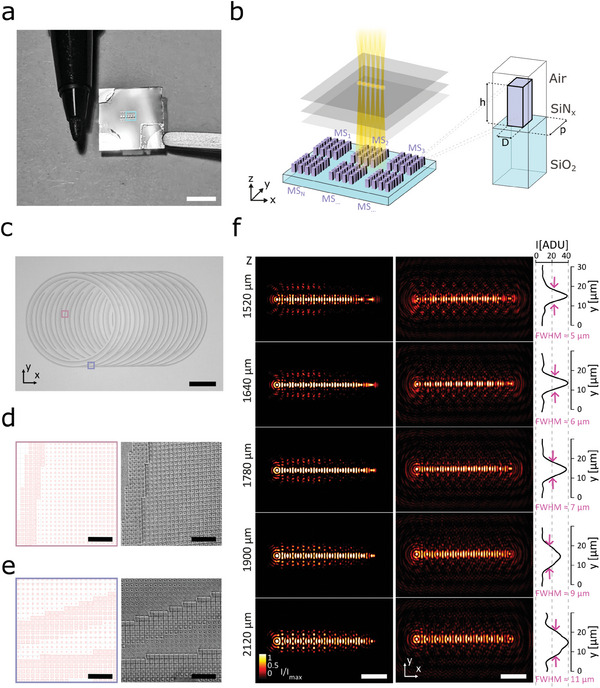
Single metasurface‐based generation of BB array – a) Example of one fabricated chip with the metasurfaces (MSs). The MS structures appear in reflectance as light‐grey stripes on the chip surface. b) Schematic of the unit nano‐pillar of the metasurfaces (MSs). c) A wide‐field image of one of the metasurfaces for Bessel beam lattice (BBL) excitation. The MS shows the characteristic rings of our BBL principle: each ring simultaneously behaves as both a converging lens and annular mask, transforming each quasi‐Delta‐Ring into a quasi‐Bessel beam. d) Magnified view of the marked central region in (c): MS layout (left) and corresponding scanning electron microscopy (SEM) image (right). e) Magnified view of a marked peripheral region in (c): MS layout (left) and corresponding SEM image (right). The SEM images show the nanometric scaling of the square nano‐scatter cross‐section. f) Simulated beam profiles of the BBL generated by our MS layout at various propagation depths (left); experimentally measured beam profiles of the BBL generated by our fabricated MS (right) and their corresponding intensity profiles along the *Y* axis. Experimentally measured full width at high maximum (FWHM) of the BBL Y‐profiles of <7 µm. Scale bars: 0.5 cm (a), 50 µm (c,f), 2 µm (d,e).

In principle, the proposed method to synthesize BBs and BBLs is compatible with different technologies for the modulation of the wavefront, i.e., traditional Fresnel optics and spatial light modulators (SLMs) encoding the corresponding diffractive pattern. Here, to assess the effective capabilities of this method, we proceeded with its fabrication adopting Meta‐Surface (MS) technology. This approach uses arrays of nanopillars a few hundred of nanometers high to control the properties of the electromagnetic field.^[^ ^28^, ^29^, ^30^, ^31^ ^]^ MSs can be used to simultaneously control the amplitude, the phase, and the polarization of a propagating light wavefront, thus enabling color routing,^[^ ^32^, ^33^ ^]^ polarization‐multiplexing,^[^ ^34^, ^35^, ^36^ ^]^ and focusing.^[^ ^37^ ^]^ For our implementation, we opted for a design based on geometrical phase correction and considered a set of possible materials for the fabrication. We selected silicon nitride (SiN_x_), a dielectric material compatible with CMOS fabrication processes,^[^ ^38^, ^39^ ^]^ with good chemical and thermal stability,^[^ ^40^, ^41^ ^]^ with high index contrast compared to the oxide cladding (Δ*n* ≈ 0.5), and with a broadband transmission extended to the visible spectrum.^[^ ^38^, ^42^ ^]^ Therefore, we fabricated and tested a series of polarization‐insensitive SiN_x_ Huygens MSs designed to operate at λ  =  478 *nm* for producing multiple BBs (**Figure** **2**a).

Our MS implementation presents a series of nanopillars, built on top of a Silicon dioxide substrate (Figure 2b), with a different lateral dimension to locally modulate the phase of the incoming wavefront, hence acting as a phase‐delay element. We used finite element numerical simulations (COMSOL Multiphysics, Experimental Section; Note S2, Supporting Information) to study the electromagnetic field modulation of the nanopillar and identified a library of 28 structures ensuring the coverage of the phase range ϕ  = [0,  2π]  with a transmittance efficiency greater than 50% (Figure S1, Supporting Information). These nanopillars present a square cross‐section with a variable side length D, ranging between 60–340 nm at steps of 10 nm, and with a fixed height *h*  =  520 nm (Figure 2b). The designed MS active area resulted in ≈200 µm in diameter with nanopillars distributed with a fixed spatial period (*p*  =  400 nm). Then, we developed a MATLAB routine to convert the continuous phase profile obtained with the Method‐S2 (Supporting Information) to the phase discretization required for the MS (Note S3, Figures S2, and S3, Supporting Information). The numerical simulations confirmed that the adopted MS layout generates a set of ten Bessel beams in close agreement with the theoretical model (Figure S4, Supporting Information). Based on this confirmation, we fabricated on the same substrate a series of MSs (MS_1_, MS_2_, …, MS_N_) (see Figure 2c**–**e, Experimental Section; Note S4, Figures S5–S8, Supporting Information).

We characterized the intensity profile generated by the MS and confirmed that we obtained a BB lattice extending over the lateral dimension X of ≈200 µm, with ten BBs with ≈ 4 × 7 × 700 µm^3^ (*FWHM_xyz_*) size each, close to the values expected from the simulations (Figure 2f; Videos S1–S3, Figures S9 and S24, Supporting Information).

As shown in Figure 2, the focal cross‐section of the Bessel beam lattice (BBL) is characterized by an array of spots that are not perfectly circular. This is the result of a process aimed at the minimization of the side lobes according to the formula reported in the previous paragraph. This result is qualitatively in good agreement with the shape of the square lattice previously reported in the literature.^[^ ^16^ ^]^

Within the metasurface characterization process, we measured the transmittance efficiency of the metasurface generating the Bessel beam lattice (BBL) as the power at the focal plane (*z* = 1900 µm) divided by the transmitted power at the metasurface plane (*z* = 0 µm) through an aperture of the same area. At the focal plane, the fabricated BBL metasurface exhibits a transmittance efficiency of 67% and a signal‐to‐noise ratio higher than 20:1, sufficient for achieving single‐cell resolution as required in the biological application demonstrated in this work.

It is worth noticing that a single Bessel beam (BB) generated with an axicon approach has a higher transmittance efficiency than a BB generated with a delta‐ring approach.^[^ ^43^, ^44^ ^]^ Indeed, axicons can convert a larger portion of the input beam into a Bessel beam compared to a delta‐ring approach. However, there are two main aspects that motivated us to use a delta‐ring approach for the generation of an array of Bessel beams.

First, axicons present more limited control over beam parameters: for example, the focal position of a Bessel beam generated with an axicon approach is limited to lie close to the axicon's surface, i.e., at the half point of the range of the BB which is formed immediately after the axicon surface.^[^ ^45^, ^46^ ^]^

Second, as proposed in this work, the area inside the delta‐ring, wasted when implementing a single delta‐ring, can be used to encode other Bessel beams, thus increasing the overall transmittance efficiency compared to other approaches which exploit only some portions of the single delta‐ring for multiplexing.^[^ ^16^, ^22^ ^]^

### 2.4 Metasurface‐Based BB‐Excitation for Light Sheet Functional Imaging of Zebrafish Neuronal Activity

To demonstrate our approach and its implementation for a fluorescence imaging application, we integrated the metasurface (MS) fabricated for the generation of a linear lattice of BBs into the excitation path of a custom light sheet microscope.

**Figure 3:**
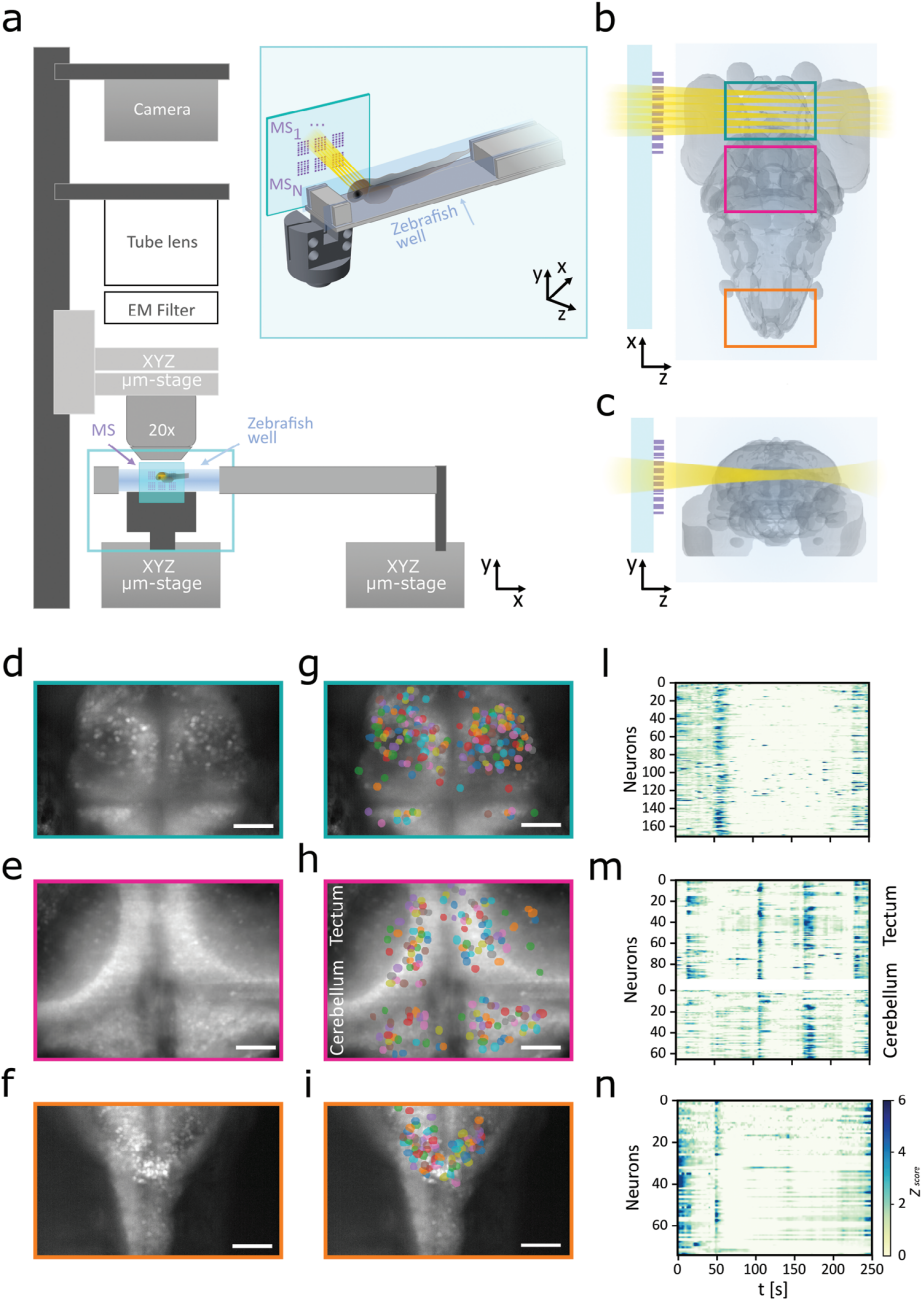
MS‐based Bessel beam lattice light sheet imaging of the zebrafish brain activity – a) MS‐based BB lattice light sheet microscope. The setup is made by two independent arms: the horizontal illumination modulus, where the BB lattice light sheet is generated by the metasurface (MS) system, and the vertical imaging column, where the fluorescence emission signal is collected by a custom‐made up‐right microscope. The zebrafish is embedded in agarose inside a millimetric 3D‐printed well. The MS chip is mounted parallel to the sagittal plane of the zebrafish with a custom‐made precision holder (cyan insert in a). b) Horizontal view of the zebrafish larva brain. The colored boxes refer to the areas imaged with the MS‐based lattice light microscope. c) Coronal view of the zebrafish larva brain. d–f) Maximum intensity projection of the brain regions highlighted in b. g–i) Segmented neurons extracted from the zebrafish brain regions highlighted in b. l) Raster plots showing the calcium activity profiles of the identified neurons of the region shown in g. m) Raster plots showing the calcium activity profiles of the identified neurons of the region shown in h. n) Raster plots showing the calcium activity profiles of the identified neurons in the region shown in i. Scale bar: 50 µm (d–i).

The MS was inserted between the laser source and the sample, in place of the optics and the objectives typically required for the LSM excitation beam (see **Figure** **3**a; Figure S8, Supporting Information). We tested our system by recording neuronal activity from zebrafish larvae expressing the genetically encoded neuronal activity reporter GCaMP6s in the majority of the neurons in the brain. The average brain size of a zebrafish larva at 6 days spot fertilization is ≈500 × 350 × 250 µm^3^ (*X*, *Z*, and *Y* directions), and present neurons whose typical diameter is 6–7 µm in diameter.

To match the conditions necessary for neural activity recording, we designed our BBL metasurface to be able to illuminate a plane of the zebrafish larva with a light sheet thickness close to the typical cell diameter and a longitudinal extension higher than the lateral brain dimension (350 µm).

As shown in the previous paragraph, we fabricated a metasurface generating a BBL with an overall *XZ*‐extension of ≈200  × 700 µm^2^ *FWHM_xz_*, and with a BBL thickness of ≈7 µm within a 350 µm‐range along the beam propagation direction (i.e., the diffraction‐free range lies from the position Z equal to 1520 µm and Z equal to 1870 µm).

This type of tissue presents a rather large degree of optical inhomogeneity and is characterized by a number of optical path mismatches, leading to a relevant level of light scattering. Nevertheless, the adopted fluorescence‐based reporter, changing its fluorescence emission as a function of the neuronal action potential firing rate, allows to reconstruct the spatial patterns of the neuronal activity at cellular resolution. For recording brain activity, the MS was aligned parallel to the sagittal plane of the zebrafish brain (see Figure 3a and Experimental Section), so to generate a BB‐lattice excitation pattern on a large area of the brain along the medio‐lateral direction (≈300 × 300 µm^2^). By means of an orthogonal detection arm equipped with a long working distance detection objective and a CMOS camera, we successfully recorded neuronal activity in multiple brain regions (Figure 3b) characterized by different amounts of light scattering, like the Pallium (Figure 3d), Tectum Opticum and Cerebellum (Figure 3e), and Medulla Oblungata (Figure 3f).

Zebrafish larval brain presents cells with 6–7 µm typical diameter and a degree of neuronal packing with almost no extracellular space. Even in these challenging conditions and using a long working distance detection objective with a limited numerical aperture (0.4NA, with respect to those commonly employed in LLS microscopy, 0.9‐1.1NA, with an expected reduction in lateral resolution of factor 0.5 and 0.16 in the axial direction),^[^ ^16^, ^47^, ^48^ ^]^ the quality of the imaging allowed us to segment the area of the individual neurons by means of an automated segmentation analysis pipeline commonly used for this application and retrieve the subtle changes of the basal fluorescence level reporting the neuronal activity (Figure 3g–i and Experimental Section).^[^ ^49^ ^]^ The reconstructed temporal series of the neuronal firing associated with spontaneous activity in the brain presented either broad spatial patterns of activity involving large populations of cells or segregated clusters of activity in small neuronal groups (Figure 3l,m; Video S5, Supporting Information). Thus, the obtained dataset confirmed the possibility to use our BB lattice generation method on a challenging application, like fluorescence‐based functional brain imaging.

The proposed BB‐LLS microscope, equipped with a 0.4NA 20× imaging objective, achieved single‐cell resolution (≈5 µm × 5 µm in the *XZ* plane) imaging the neuronal activity (GCaMP6s emission peak at the wavelength value of λ ≈510 nm) in a living organism.

More information on the experimental ethical approval and on the BB‐LLS microscope can be found in the Experimental section.

## 3 Discussion

Combining methods of wavefront modulation and nanofabrication, we integrated in a single sub‐millimetric surface all the optical operations required to generate at the sample space a group of Bessel beams for light sheet microscopy. While current reports focus on methods for generating BB‐arrays characterized by regular geometrical patterns known as lattices, we adopted a beam tiling strategy suitable for a small‐footprint LSM configuration, capable at the same time of extending the actual flexibility in the design of BB‐arrays and achieving more precise and independent control of all the features of the individual beamlets. Indeed, the focal position, the XY‐position, and the depth of field of each BB within the array can be independently designed, potentially enabling the generation of envelopes of BBs with an arbitrary 3D structure along the light propagation direction and arbitrary extension in the perpendicular plane, and so going beyond the regular lattice geometry. With respect to the current state of the art, the proposed approach presents several advantages. First, by embedding all the optical operations necessary to generate a BB lattice in one single optical element, we show that it is possible to substantially reduce the footprint and the complexity of the traditional microscopy setups for life science research. This has the potential to dramatically boost the level of integration and miniaturization of optical systems. Second, since the lateral extension of the light sheet generated with this method is independent of the numerical aperture of the lattice light sheet focusing lens, this approach can be used to generate a lattice light sheet with millimetric lateral extension without compromising the light sheet thickness; hence, for a given light sheet thickness, it is possible to generate a lattice light‐sheet with a lateral extension far beyond the current state‐of‐the‐art. Third, taking advantage of the “in‐plane” beam multiplexing, our approach allows for maximizing the light utilization efficiency. Indeed, it is common in traditional LLS designs to block the input beam light at the center of the apodization mask while, in our design, the central part of the input beam contributes to the generation of other BBs in the lattice. The single plane strategy we devised for the generation of BB lattice could be in principle embodied using different technologies for the fabrication of flat optics, like traditional Fresnel Lenses and DOEs, or using devices for dynamic modulation of the light wavefront, like SLMs. We adopted here MS technology as it supports the simultaneous but independent modulation of different parameters of the electromagnetic field, like amplitude, polarization, and phase^[^ ^20^, ^37^, ^50^ ^]^ and it allows the potential to exploit their tunability and reconfigurable properties.^[^ ^51^, ^52^ ^]^ With respect to traditional flat optics and DOEs, MS technology offers today implementations ensuring more than one simultaneous operational wavelength (e.g., achromatic and broadband metalenses)^[^ ^31^, ^53^, ^54^ ^]^ and compatibility with high‐index‐contrast (HIC) materials. On the other side, while SLMs do offer advantages in terms of dynamic tunability of the phase profile and intrinsic correction for operating at different wavelengths, compared to MS, they are still limited in the actual spatial frequency resolution deriving from the pixel‐to‐pixel crosstalk that dramatically impacts on the device diffraction efficiency uniformity. Moreover, the sub‐millimetric size of the optical elements designed according to the proposed strategy allows for the fabrication on the same substrate of multiple lens layouts, each with optical properties tailored around different requirements, making the fabricated optical element a flexible and versatile multi‐scale and multipurpose imaging system. Using the metasurface technology, we therefore fabricated an optical element in a total area smaller than one by one squared millimeter capable to generate a designed intensity distribution over an active area of 200 µm in diameter. Its successful integration within an optical path for light sheet excitation microscopy allowed us to validate the novel optical component in a challenging application: the recording of the neuronal activity from the zebrafish larva brain at cellular resolution. This demands taking into account a number of aspects: i) generation of excitation beams with extreme aspect ratios, with longitudinal extensions covering hundreds of micrometers (to match the size of the zebrafish brain at the larval stage, ≈300 µm) and transversal thickness of a few microns (close to the typical neuronal cell diameter, ≈6 µm); ii) with respect to in vitro conditions, this application scenario is substantially more affected by light scattering originating from the different cellular components packed into the tissue with different density, homogeneity, and optical properties; iii) brain functional recordings, like those reported here, rely in the capability to detect subtle variations of the fluorescence emission with respect to baseline levels, requiring excitation and detection configurations optimizing the acquisition signal‐to‐noise ratio. We show here that all these constraints are satisfied also with the compact optical approach based on the MS we developed.

In conclusion, our design and MS‐based integration open a range of possible application scenarios of BB lattices not only in microscopy development but more in general in all those fields where small‐footprint methods for light shaping with multiple BBs are instrumental for increasing the parallelization, the throughput and resolution levels.

## 4 Experimental Section

### Numerical Simulations of the Nano‐Pillars Electromagnetic Response

The electromagnetic response of individual nano‐scatter must be engineered such that their phase parameter space satisfies the target phase ϕ(*x*,  *y*). This implies identifying i) a set of nano‐scatters that can accumulate a difference in phase thanks to either their geometry or orientation^[^ ^37^ ^]^ while considering ii) all the possible fabrication limitations (e.g., maximum spatial resolution, minimum period gap‐size, etc.). To predict the phase accumulated by each nano‐pillar and their transmittance COMSOL Multiphysics platform was used which is widely used for finite element method (FEM) numerical simulations of optoelectronic and photonics devices (see Video S4, Supporting Information). The results of transmittance and phase of each nano‐pillar are described in Note S2 and shown in Figure S2 (Supporting Information). The algorithm developed for the metasurface design wastaken as input the nano‐pillar discrete phases and used them to reconstruct the analytical phase profile. To test the performance of the metasurface phase profile the beam propagation was characterized experimentally and compared with the simulated beam propagation.

### Metasurface Design, Fabrication, and Simulation of the Expected Performance

To design the metasurface, to create the layout required for the fabrication, and to simulate its performance, a semi‐automatized MATLAB routine (see Note S3, Figures S2 and S3, Supporting Information for a detailed description of the MATLAB pipeline and of the BPM) was implemented.

The SiN_x_ metasurfaces were fabricated at the EPFL Center of Micro‐Nanotechnology (CMi). In the last years, these features had made SiN_x_ manufacturing technology more and more accessible for micro‐nanofabrication of optical components for imaging applications.^[^ ^55^, ^56^ ^]^

First, a 520nm‐thick silicon nitride (SiN_x_) layer was deposited on the silicon dioxide (SiO_2_ – 525 µm thick) as core material. Second, to create the MS pattern, we spatter 30 nm of chromium (Cr) as a hard mask and as an opaque reflective layer for proper focusing of the ebeam‐tool and we spin coat 200 nm of hydrogen silsesquioxane (HSQ) as a positive resist for the e‐beam lithography. The MS pattern was thus created on the photoresist after development. The MS structure was then transferred into the Cr and SiN_x_ layer by one first step of ion beam etching (IBE) followed by a high‐density inductively coupled plasma (ICP) etching step based on fluorine chemistry (CHF_3_/SF_6_). Finally, the residual of Cr and photoresist is stripped by wet acid etching. The main fabrication steps are depicted in Note S4 and Figure S5 (Supporting Information). Images of the fabricated chips are shown in Figure 2c–e; Figures S6 and S7 (Supporting Information). Finally, the same nano‐pillar library and fabrication process flow can be used to generate phase profiles such as the phase profile of a cylindrical lens or of a single Bessel beam (see Note S7, Figures S18 and S19, Supporting Information).

It is worth mentioning that in the current metasurface layout, the area around the metasurface was not masked and part of the input beam could be transmitted through the chip without being focused by the metasurface. Therefore, to increase the contrast, one should shape the input beam to fit the MS aperture.

### Metasurface‐Based Bessel Beam Characterization

The profile of the Bessel beam lattice light sheet was measured by acquiring its intensity along the propagation direction. For these characterization measurements, a 20× Olympus objective was mounted with a kinematic mount on a one‐axis motorized stage (PT1/M‐Z8, Thorlabs). The objective was oriented parallel to the propagation beam and adjusted such to focus on the chip surface that we defined as the zero‐propagation‐position, *z* = 0 µm (Figure S8, Supporting Information). The metasurface chip was fixed in the vertical slit of a custom‐made rotating holder mounted on a three‐axis stage to align its surface perpendicular to the incoming beam. The BB light sheet profiles were acquired at different propagation positions *z*, from z_0_ = 500 µm up to z = 3500 µm with a step size of z_step_ = 5 µm with a CMOS camera (XIMEA, MQ013RG‐ON) at an exposure time of 1 ms. All the metasurface characterization measurements were performed with a 488 nm Laser (Cobalt 06‐01) at a laser power value of P_w_ ≈0.3 mW. The BB‐LLS thickness was then estimated by measuring the full width at half maximum (FWHM) of the X and Y intensity profile at a depth ranging from 1500 µm up to 2200 µm every 120 µm.

### Metasurface‐Based BB‐LLS Microscope

The metasurface chip for BB‐LLS illumination is integrated into a custom up‐right microscope thanks to two mechanical holders we designed to fit the required physical constraints imposed by the MS chip, the sample holder, and the imaging objective (Figure 3a; Figures S20 and S21, Supporting Information). The MS chip holder can be easily placed into the XYZ‐stage (a three‐axis stage, MBT616D/M, Thorlabs) to enable the aliment of the MS with the input beam (operating wavelength λ ≈488 nm; 488 nm‐Cobolt‐06‐01 Laser). The sample holder was mounted on a second identical stage to adjust the sample sagittal plane parallel to the chip surface. Emitted light from the sample was collected by the air objective lens (MPLAN APO20 20×/NA0.42 Mitutoyo), filtered (MF510‐42 – WGFP Emission Filter, CWL = 510 nm, BW = 42 nm), and then imaged by a tube lens (fTL = 200 mm) onto the CMOS camera (XIMEA, MQ013RG‐ON).

### Sample Preparation for Live Imaging

Zebrafish brain imaging experiments were carried out in line with the current Italian legislation (Decreto Legislativo 4 Marzo 2014, n.26) and were approved by the Ethical Committee of the University of Padua (61/2020_dalMaschio) and adhere to the ARRIVE (Animal Re‐search: Reporting of In Vivo Experiments) guidelines.

Larvae were raised at 28 °C on a 12h light/12h dark cycle using standard procedures. Elavl3:H2B‐GCaMP6s larvae were used for brain imaging. Five days post‐fertilization (dpf) larvae were embedded in 2% low melting point agarose gel on a 3D printed custom‐made support (Figure S20, Supporting Information).

### Zebrafish Brain Imaging and Data Analysis

The microscope and all components were controlled with µManager.^[^ ^57^ ^]^ The 488 nm laser output power was set to deliver ≈1 mW at the sample. The brain of the embedded living zebrafish was moved into focus using the coarse and fine‐focusing screws of the translation stage. The spatial and temporal resolution of the image stack acquisition were 0.240 µm/pixel and 500 ms per frame, respectively. Spontaneous activity was recorded for 250 s from different brain regions and with different subjects.

The recorded raw movies of the brain regions were then processed using Suite2p^[^ ^49^ ^]^ for automatic segmentation of the active neurons and to extract their calcium activities.

## Conflict of Interest

The authors declare no conflict of interest.

## Author Contributions

Research design was carried out by A.A. and M.d.M. Conceptualization was done by A.A. Microscope design and setting‐up was done by A.A. Microscope operation was carried out by A.A. and M.B. Metasurface design was done by A.A. Metasurface fabrication was done by A.A. Metasurface characterization was done by A.A. Image analysis was performed by M.B., A.A., M.d.M. Transgenic zebrafish handling and preparation was carried out by M.B. and M.d.M. Mathematical derivation of the method was done by A.A. MATLAB code was performed by A.A. Methodology was developed by A.A., M.B., G.T., and M.d.M. Investigation was performed by A.A. and M.d.M. Visualization was carried out by A.A., M.B., G.T., and M.d.M. Supervision was done by G.T. and M.d.M. A.A. wrote the original draft. A.A., M.B., G.T., and M.d.M. wrote, reviewed and edited the final drafts.

## Supporting information



Supporting Information

Supplemental Video 1

Supplemental Video 2

Supplemental Video 3

Supplemental Video 4

Supplemental Video 5

## Data Availability

The data that support the findings of this study are available on request from the corresponding author. The data are not publicly available due to privacy or ethical restrictions.
