# Supplementary material for: A Single Sub‐Millimetric Metasurface‐Based Optical Element for Lattice Bessel Beam Excitation Enabling Brain Activity Recordings In Vivo
Source: Small. 2025 Feb 2;21(9):2409258. doi: 10.1002/smll.202409258 (PMC11878255; doi:10.1002/smll.202409258)
Supplement: Supplementary file 1 — Supporting Information [file SMLL-21-2409258-s005.docx]

Supplementary material for

A single sub-millimetric metasurface-based optical element for lattice Bessel beam excitation enabling brain activity recordings in vivo

Anna Archetti *et al.*

* Corresponding authors: [anna.archetti@unipd.it](mailto:anna.archetti@unipd.it), [marco.dalmaschio@unipd.it](mailto:marco.dalmaschio@unipd.it)

This file includes:

- Supplementary Notes 1-7
- Supplementary Figures 1-24
- Supplementary Table 1-2,
- Supplementary References

Other supplementary material includes:

Videos from S1 to S5

# Supplementary NOTES

S1. Design of the phase profile of a Bessel beam array

The first approach (method-1) for the generation of the phase profile of a Bessel beam array relies on the following main steps.

**Step (1)**: the phase profile $\phi_{j}$ of a convergent lens with the desired radius $R_{j}$ and focal length $f_{j}$ is defined by

$$\phi_{j}\left( x,y \right)=-\frac{2\pi}{\lambda}\left[ \sqrt{\left( x-P_{j} \right)^{2}+\left( y-Q_{j} \right)^{2}+{f_{j}}^{2}}-f_{j} \right]; x, y\in\mathbb{R}^{2} :x, y\leq R_{j}$$

where j is an integer number: $j=1, 2, ..N$ to index the beam and $N$ is the desired total number of beams in the array. $P_{j}$ and $Q_{j}$ are two spatial parameters defining the position of the beam -j. The values of $R_{j}$, and $f_{j}$ set the desired focal position and the effective numerical aperture (${NA}_{j}$) and therefore the maximum size of point spread function (PSF) of the beam *j* in the lateral (XY) and longitudinal (Z) directions.

**Step (2)**: the annular mask $M_{j}\left( x,y \right)$ is defined by:

$$M_{j}\left( x,y \right)=\left\{ \begin{aligned} 1, &R_{j}-\Delta_{j} \leq\sqrt{({x-P_{j})}^{2}+{(y-Q_{j})}^{2}}\leq R_{j} \\ 0, &elsewhere \end{aligned} \right.$$

with external diameter $R_{j}$ and thickness $\Delta_{j}$, and where radius $R_{j}$of the ring corresponds the radius defined for $\phi_{j}\left( x,y \right)$ in step 1. For a given NA, the smaller $\Delta_{j}$, the lower the beam thickness and the longer its depth of focus.

**Step (3)**: the phase profile $\phi_{j}$ is multiplied by the mask $M_{j}$ such to obtain the phase profile:

$$\phi_{ring, j} \left( x,y \right)=\left\{ \begin{aligned} -\frac{2\pi}{\lambda}\left[ \sqrt{{(x-P_{j})}^{2}+{(y-Q_{j})}^{2}+{f_{j}}^{2}}-f_{j} \right], &R_{j}-\Delta_{j} \leq\sqrt{({x-P_{j})}^{2}+{(y-Q_{j})}^{2}}\leq R_{j} \\ 0, &elsewhere \end{aligned} \right.$$

**Step (4)** the field distribution is $\boldsymbol{E}_{\boldsymbol{j}}\boldsymbol{=}\boldsymbol{E}_{\boldsymbol{0}\boldsymbol{j}}\boldsymbol{e}^{\boldsymbol{i}\phi_{ring, j}}$ of an electromagnetic wave characterized by a phase profile $\phi_{ring, j}$ equal to the phase profile obtained in step (3) and with amplitude $E_{\boldsymbol{0,j}}$ corresponding to the annular mask:

$$E_{0j}\left( x,y \right)=\left\{ \begin{aligned} 1, &R_{j}-\Delta_{j} \leq\sqrt{({x-P_{j})}^{2}+{(y-Q_{j})}^{2}}\leq R_{j} \\ 0, &elsewhere \end{aligned} \right.$$

**Step (5)**: **Step (1)** to **Step (4)** are repeated for *N* times corresponding to the desired number *N* of beams in the array. At each iteration *j*, the phase profile can be designed with the desired focal length $f_{j}$, ring thickness $\Delta_{j}$ and ring radius $R_{j}$. The ring position can be shifted of the desired parameters $P_{j}$ and $Q_{j}$.

**Step (6)**: the total phase profile $\phi_{TOT}$ is computed as the result of the argument of the sum of the fields $E_{\boldsymbol{j}}$ each corresponding to one Bessel beam shifted in space of the desired parameters $(P_{j}, Q_{j}$). Therefore, the final phase profile is $\phi_{TOT}=arg\left\{ \sum_{j=1}^{N} E_{j} \right\}$ where $j=1, 2, ..N$ and $N$ is the total number of rings.

The second approach (method-2) for the generation of the phase profile of a Bessel beam array relies on the following main steps:

**Step (1)** to **Step (3)** the same as in method-1.

**Step (4)**: step (1) to step (3) are repeated for *N* times corresponding to the desired number *N* of beams in the array. At each iteration *j*, the phase profile $\phi_{ring, j}$ can be designed with the desired focal length $f_{j}$, ring thickness $\Delta_{j}$ and ring radius $R_{j}$. The ring position can be shifted of the desired parameters $P_{j}$ and $Q_{j}$.

**Step (5):** the values of the total phase profile $\phi_{TOT}(x,y)$ are the values of each single ring $\phi_{ring, j}$ and in all points $(x,y)$ belonging to multiple rings, $\phi_{TOT}(x,y)$ always takes the value of the phase of the ring $\phi_{ring, j}$ with minimum index.

Therefore, in this case the total phase profile $\phi_{TOT}(x,y)$ is defined as:

$$\phi_{TOT}\left( x,y \right)=\phi_{ring, j}\left( x,y \right)$$

where j is the natural number minor or equal to N, defined by

$$j\left( x,y \right)=\min k :R_{k}-\Delta_{k} \leq\sqrt{({x-P_{k})}^{2}+{(y-Q_{k})}^{2}}\leq R_{k}$$

NB. The number of modulated patters must be $N<2R/\Delta$ to avoid significant loss of phase information. (In this work, $R=100 \mu m$, $\Delta=17 \mu m$ (See Discussion for a further discussion on these methods).

We highlight how, among these two approaches, the first (method-1) is definitely the more efficient: it allows the generation of arrays of Bessel beams with arbitrary 3D geometry and optimal uniformity. On the other hand, method-2 is more limited, as it allows to generate linear arrays of Bessel beams with particular values of the period P, close to the ring thickness, and with a number of beams close to the ratio of the ring radius and the ring thickness. Although the latter method generates an array which is uniform only in the central part and with less geometrical flexibility, it has been our choice for the demonstration of the approach and of fabrication process because it was the easiest to implement. Indeed, in the development of a new fabrication process the presence of a macroscopic pattern repeating itself over the entire chip surfaces greatly simplifies both the masks generation and the real-time production quality verification, especially for non-uniformity issues which frequently happen during the costly e-beam lithography step performed on a custom chip (instead of on a standard wafer).

In this work our goal was twofold: to demonstrate a proof-of-concept of the methodology, and to show its application on a live organism. We therefore decided to limit the demonstration to simpler to build method-2. A further embodiment of this whole work with method-1 would have differed from the present one only for a single analytical step. Overall, once the proof-of concept has been established, we recommend method-2 only for testing new fabrication processes, and method-1 for the final application.

S2. Numerical simulation for the study of the nano-pillars response

To engineer the nano-pillars and to extract their transmittance and phase, we studied their electromagnetic response with COMSOL Multiphysics (version 5.5) which integrates the Maxwell’s equation using finite element approach:

| $\nabla\times\left( \nabla\times\boldsymbol{E} \right)-k_{0}^{2}\epsilon_{r}\boldsymbol{E=0}$ | ( 1 ) |
| --- | --- |

where $\epsilon_{r}=\left( n-ik \right)^{2}=n^{2}$ is the relative permittivity, $n$ and $k$ are the real and the imaginary part of the refractive index $n$ respectively, $k_{0}={2\pi}/\lambda$ is the wave number of free space. Each silicon nitride nano-pillar unit was simulated with a periodic boundary condition along the transverse direction with respect to the propagation of light and a perfectly matched layer and input/output ports boundary conditions along the longitudinal direction. We then extracted their transmission $T$ and phase delay $\phi$ of the electric field from the scattering parameter $S_{21}$ of the $S-parameter$ matrix, measured from the eigenmode expansion of the electromagnetic field at the output port 2 (see **Supplementary Figure 1a**). Therefore, if $E_{1}$, $E_{2}$, $E_{3}$,… are the electric field patterns of the fundamental modes on port $i$ 1, 2, 3, … , and we assume that the fields are normalized with respect to the integral of the power flow across each port cross section, then the computed mode field ($\boldsymbol{E}_{c}$) at all output port boundaries is:

$$\boldsymbol{E}_{c}={\sum_{i=1} S_{i1}\boldsymbol{E}}_{i}$$

$$\begin{matrix} \phi=- arg(E^{out})=- arg(S_{21}) \\ T=\left| E^{out} \right|^{2}=abs(S_{21})^2 \end{matrix}$$

where $E^{out}$ is electrical field at the port 2 and $S_{21}=\sqrt{\frac{Power deliverd to Port 2}{Power delivered to Port 1}}$.

We swept the geometrical parameters of the nano-pillars, till we could find a set of nano-pillar with side lengths $D$, height $h$ and period $p$, which could provide a phase coverage of 2π at an operation wavelength of 478 nm and with an average transmittance higher than 50%. As described in **Supplementary Figure 1** and **Supplementary Video 4**, we selected a set of nano-pillars with square section with a height $h=520 \mathrm{nm}$, a period $p=400 \mathrm{nm}$ and a side $D$ ranging from $D=60 \mathrm{nm}$ up to $D= 340 \mathrm{nm}$ with a side step of 10 nm. With this library of nanopillars, the target phase profile can be discretized in 28 levels.

It is worth noticing that we inverted the final sign of output phase extracted from our COMSOL simulation. We are inverting the phase and polarization because in COMSOL the phase convention is based on the following definition of the electrical field ($\boldsymbol{E}^{CM}$):

| $\boldsymbol{E}^{CM}(x,y,z, \phi)=\boldsymbol{E}_{\boldsymbol{0}}\boldsymbol{(}x, y,z\boldsymbol{)}e^{-i \boldsymbol{\phi}}$ | ( 2 ) |
| --- | --- |

Thus, while in our formalism and metasurface design an increase of the phase value indicates retardation (delay accumulation) in phase profile, in COMSOL, a phase increase introduces an electrical field anticipation.

**
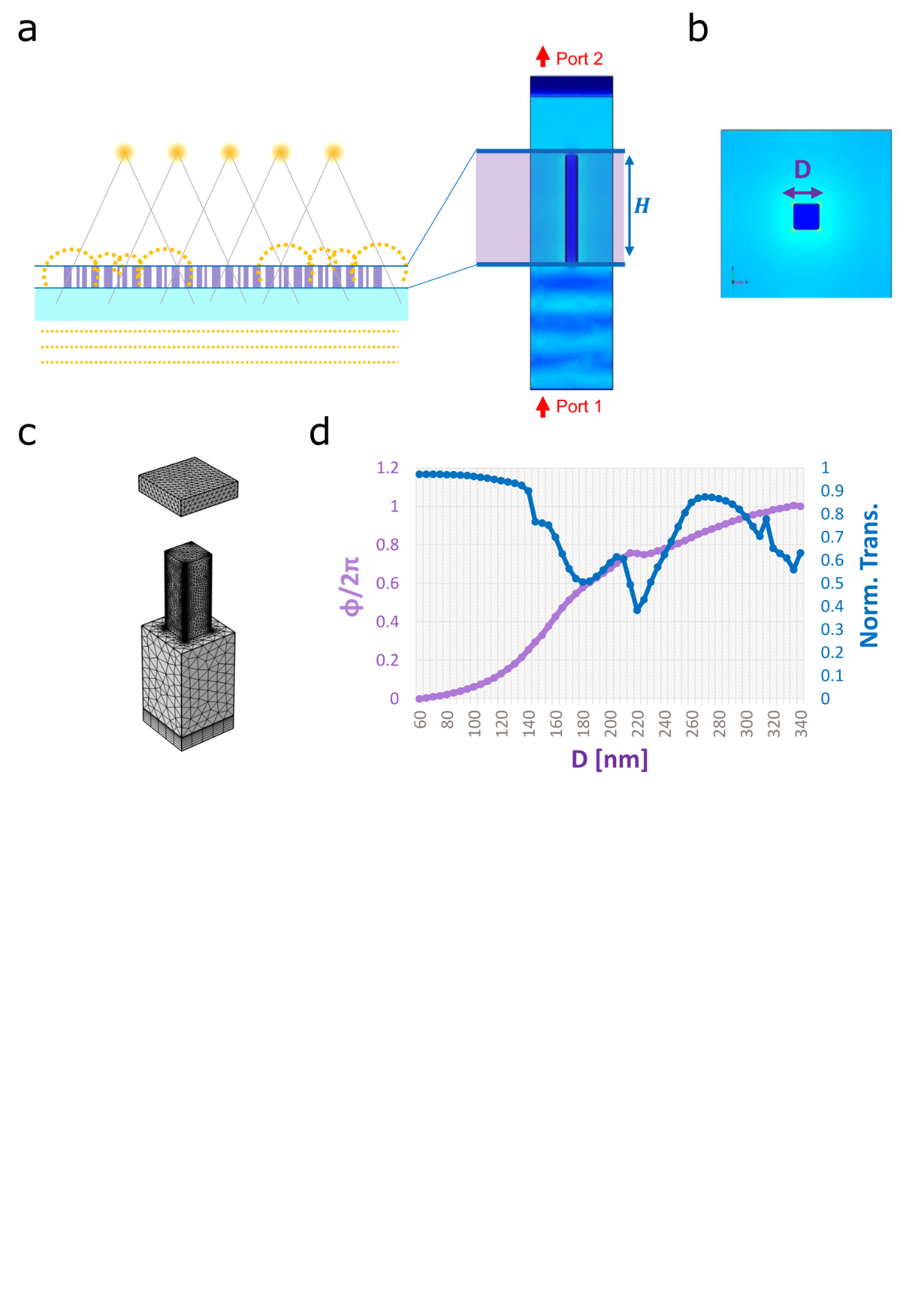
**

**Supplementary Figure 1 – Numerical simulation of the Silicon Nitride nano-pillar response. a**, Side view of the Silicon Nitride nano-pillar unit (**right** - dark blue) of the metasurface cross section (**left**). Port 1 is the input port at the interface between silicon dioxide substrate and air. Port 2 is the output port on the top of the simulated air volume surrounding the silicon nitride (SiNx) nanopillar structure. The transmittance and phase of the output electrical field are extracted via the scattering parameter $S_{21}$. **b**, Top view of the SiNx nano-pillar unit (dark blue). **c**, 3D view of the mesh generated in COMSOL to simulate the electromagnetic field modulation of each SiNx nano-pillar. **d**, Phase (violet dots) and Transmittance (black dots) of each nano-pillar simulated in COMSOL. The increase of the nano-scatter cross section (diameter D) induces an increase on the relative phase delay accumulated by the incoming light.

S3. Metasurface design and numerical simulation of the metasurface beam propagation

We developed a semi-automatized routine written in MATLAB to design metasurfaces either with a cylindrical lens profile, or a phase profile capable of generating a single Bessel beam or a lattice of Bessel beams. As described in **Supplementary Figure 2** the code is structured in four main steps. In the first step, the software loads the COMSOL input data (the phase and the transmittance values of each nanopillar of the library) and computes the analytical phase profile and numerical aperture (NA) for a metasurface characterized by the input parameters (e.g. focal length, radius, period) set by the user. The two-dimensional (2D) phase profile is then digitalized (second step) where the different nanopillar geometries are distributed over the MS surface. At this point (third step) the software generates the MS layout (**Supplementary Figure 3**) and computes quality parameters such as the error introduced on the phase with the phase discretization process. In the last step, we developed a custom Beam Propagation Method (BPM) algorithm implemented in MATLAB to calculate the rendered beam intensity profile of both the target phase profile and the discretized phase profile (**Supplementary Figure 4**). The BPM algorithm we implemented, following [1], [2], [3] and the approach reported in "Beam Propagation Method of Edgar Guevara Codina (2007)”, performs the following main steps:

(1) first it computes the Fourier transform of the electrical field $\boldsymbol{E}$ transverse to the propagation axis Z at a certain fixed position z as:

| $\hat{\boldsymbol{E}}\left( k_{x},k_{y};z \right)=\hat{\boldsymbol{E}}\left( k_{x},k_{y};0 \right)e^{\pm ik_{z}z}$ | ( 3 ) |
| --- | --- |

where $k_{x},k_{y}$ are the spatial frequencies coordinates of the Cartesian transverse coordinates x and y and $\boldsymbol{\phi}$ the phase value in the $\left( x,y, z \right)$ position. In an arbitrary image plane at the z location, the Fourier spectrum of $\boldsymbol{E}$ is equal to the spectrum in the object plane ($z=0$) multiply by the factor $e^{\pm ik_{z}z}$ where the sign ‘$+$’ corresponds to a wave propagating the forward positive direction $z > 0$ and ‘$-$‘ sign corresponds to a wave propagating in the negative half-space $z <0$.

(2) Then, the algorithm compute the inverse Fourier transform of (3):

| $\boldsymbol{E}\left( x,y, z \right)=\iint_{-\infty}^{\infty} \hat{\boldsymbol{E}}\left( k_{x},k_{y};0 \right)e^{i[k_{x}x + k_{x}x]}e^{\pm ik_{z}z}dk_{x}dk_{y}$ | ( 4 ) |
| --- | --- |

where

| $\hat{\boldsymbol{E}}\left( k_{x},k_{y};0 \right)=\frac{1}{4\pi^{2}}\iint_{-\infty}^{\infty} \boldsymbol{E}_{\boldsymbol{0}}e^{i \boldsymbol{\phi}}e^{-i[k_{x}x + k_{x}x ]}dxdy$ | ( 5 ) |
| --- | --- |

is the Fourier transform of the electrical field at the MS plane $(z = 0)$ and $\boldsymbol{\phi}$ and $\boldsymbol{E}_{\boldsymbol{0}}$ are the phase and the electrical field amplitude of each nanopillar at the position $(x, y)$ on the $z = 0$ plane respectively.

(3) Finally, for each z-position, the algorithm computes and saves in a .tiff stack the intensity of the beam as the squared of the electrical field propagation:

| $I\left( x,y, z \right)=\left\vert\boldsymbol{E}\left( x,y, z \right) \right\vert^{2}$ | ( 6 ) |
| --- | --- |

**Supplementary Figure 3** shows the MS layouts of a MS for BB LLS generation obtained with the Silicon Nitride nano-pillar geometries described in **Supplementary Note 2**. The layout is obtained starting from a phase profile of a convergent lens with focal length f = 2000 µm and diameter of 200 µm, for an operating wavelength of 478 nm.

**
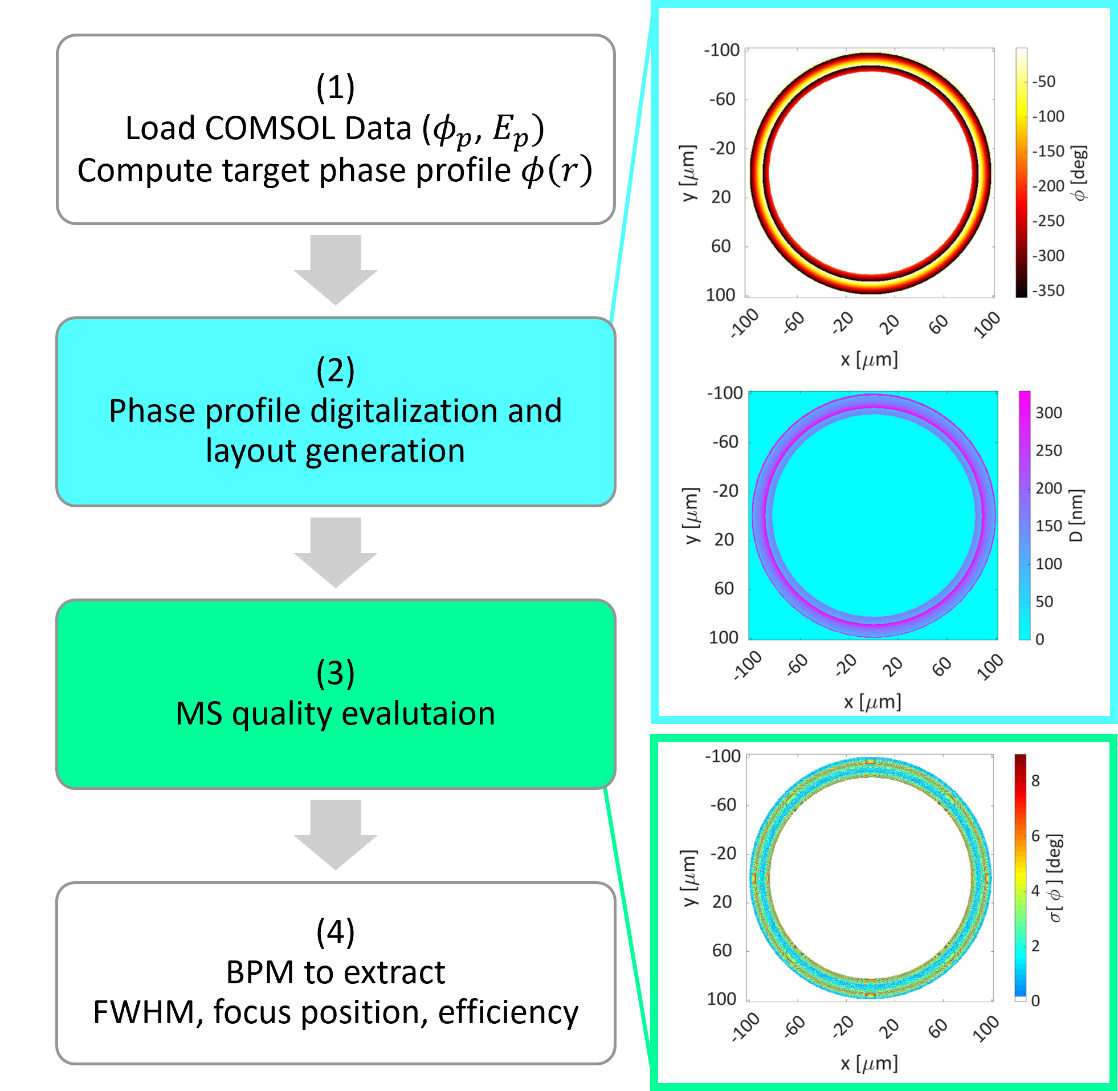
**

**Supplementary Figure 2 – Metasurface phase design. Left,** Main step of the MS design routine. First (1) the phase and transmittance of each nano-pillar simulated with COMSOL are loaded and the target phase profiles is generated. Secondly (2), the target phase profile is digitalized with the discrete parameter space of the nano-pillar phase and the MS layout is generated. Then (3) the error on the phase is calculated and displayed. Finally (4) the beam propagation is simulated and displayed with its relative quantitative information. **Right,** an example of the plots generated by the second step of the pipeline: the 2D phase profile generated with our nano-pillar library (top), the metasurface layout (center) and the error on the phase, i.e. distance from the theoretical value (bottom).


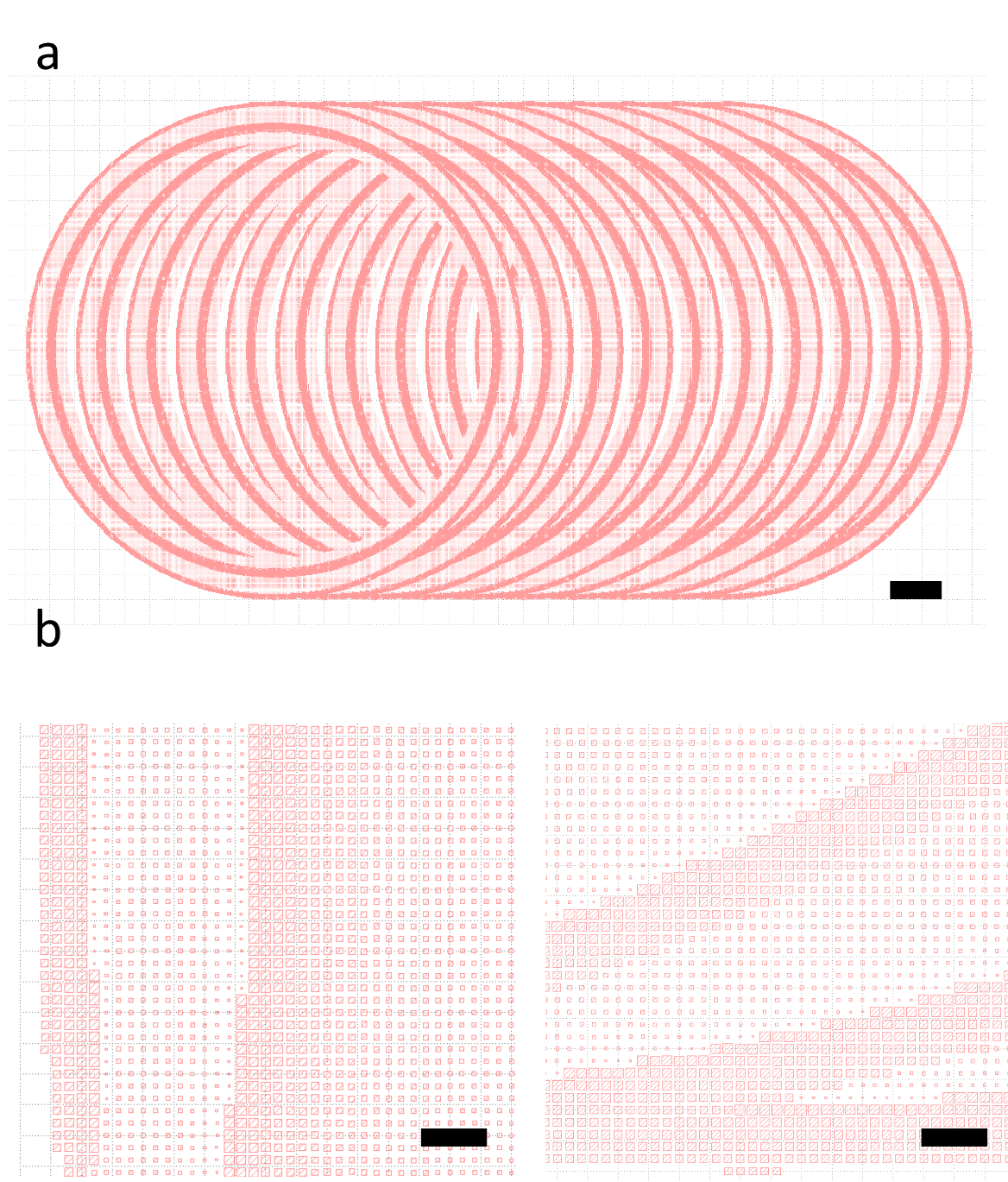


**Supplementary Figure 3 – Layout of the metasurface for the generation of**. **a**, BBL mask layout. Scale bar 20 µm. **b,** Two details of the BB-LLS mask layout showing the scaling of the cross section of the nano-pillars required to control the relative phase delay of the incoming light.

**
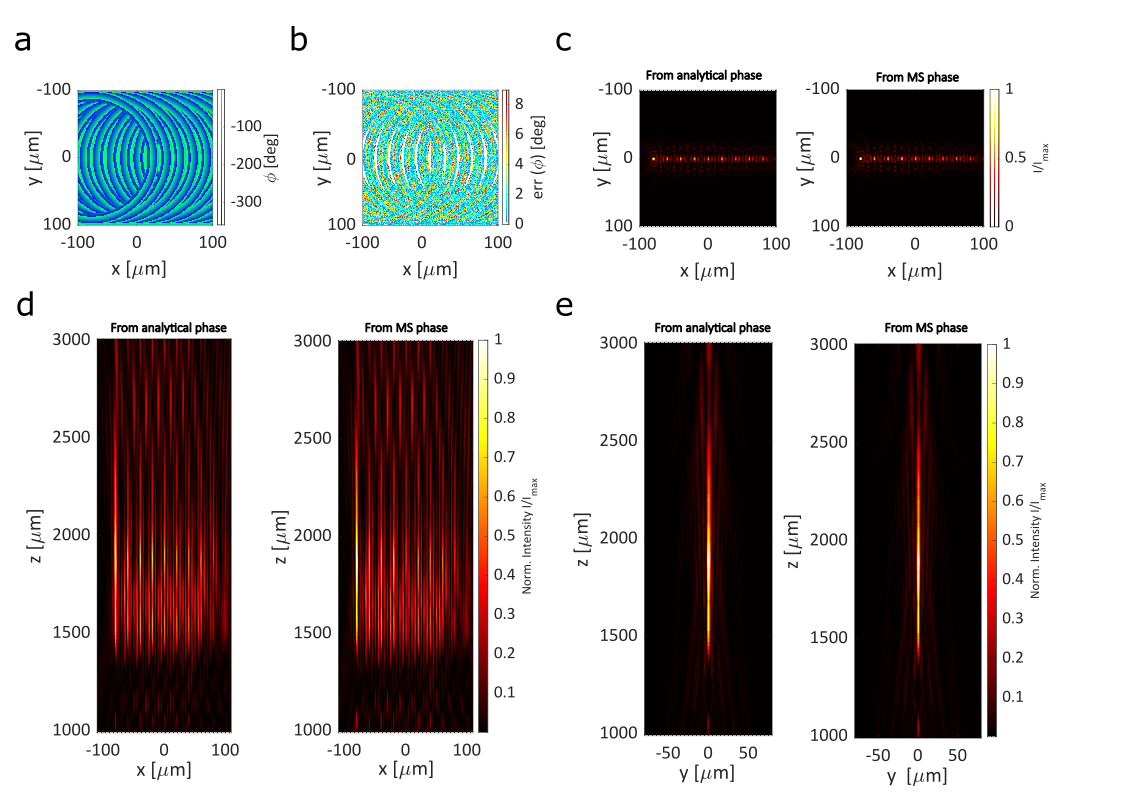
**

**Supplementary Figure 4 –** **Beam profile obtained from the analytical phase profile (method-2) and from the discretized phase profile of our metasurface achieved with 28 discretization levels.** **a,** Discretized phase profile of our metasurface (MS). **b,** Error on the phase profile. The error is computed as the distance of the discretized phase from the theoretical phase. **c,** Simulation of the XY beam profile of the BB-LLS generated with an ideal phase profile (analytical result – **left**) and with our metasurface phase profile (**right**). **d,** Simulation of the XZ beam profile of the BB-LLS generated with an ideal phase profile (analytical result – **left**) and with our metasurface phase profile (**right**). **e,** Simulation of the YZ beam profile of the BB-LLS generated with an ideal phase profile (analytical result – **left**) and with our metasurface phase profile (**right**).

S4. Metasurface fabrication and characterization

We fabricated our metasurface in the nanofabrication facilities of the Center of MicroNano Technology CMi at EPFL. The fabrication of our Silicon Nitride metasurfaces requires e-beam lithography technology to achieve up to 5 nm resolution. **Supplementary Figure 5** and **Supplementary Table 1** describe the main steps of the fabrication process. We inspected the metasurface chip surface with both optical and scanning electron microscopy as shown in **Supplementary Figure 6** and **Supplementary Figure 7**.

We systematically performed our MS characterization using a semi-automatized procedure with the setup described in **Supplementary Figure 8**. The stack of images of the beam profile focused by the MS were acquired using µManager[4] to control both the motorized z-stage and the camera. We then reconstructed the beam profile with a MATLAB-based script that we wrote to retrieve the main parameters of the beam.

**
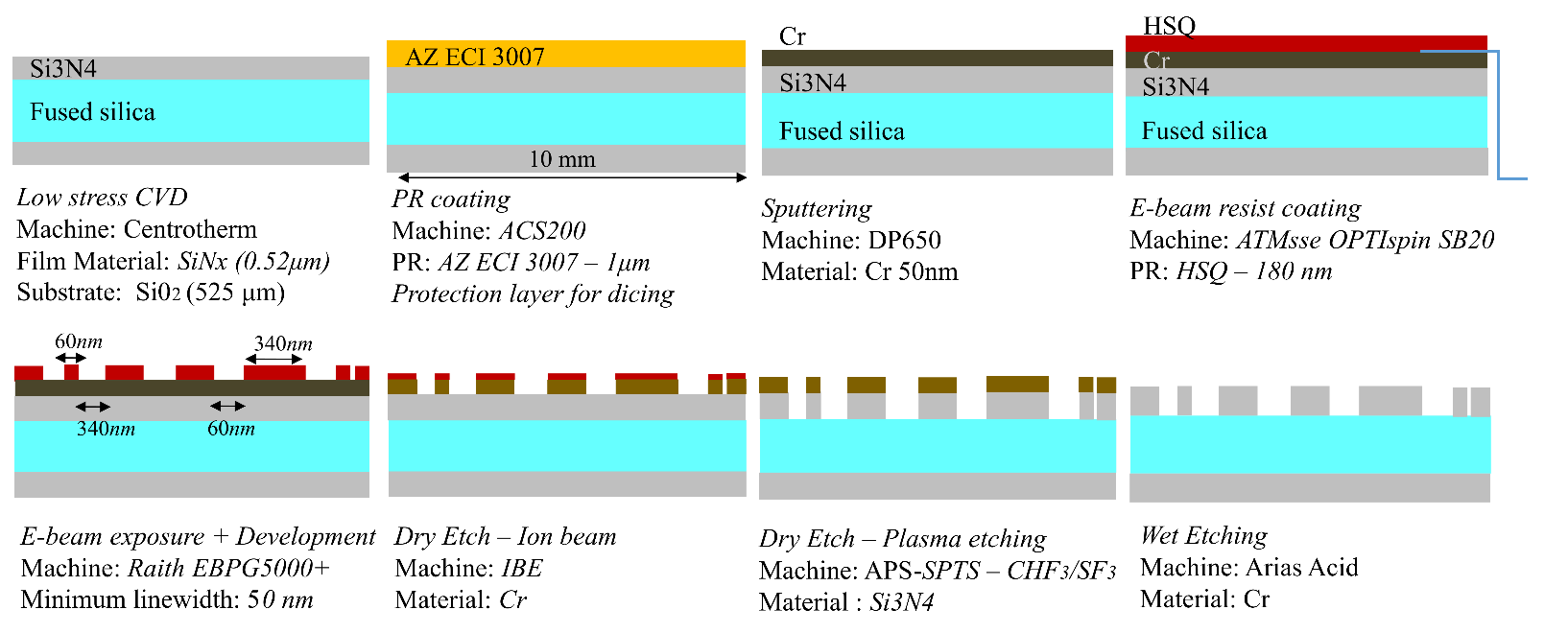
**

**Supplementary Figure 5 – Metasurface fabrication process flow**. A 520 nm thick Silicon Nitride film is deposited with a Chemical Vapor Deposition process (CVD) on a 525 µm-thick Silicon dioxide substrate. A 1.5 µm thick AZ ECI 3007 resist film is spin coated on the SiNx surface as protective layer for the wafer dicing step. After a cleaning step to remove the resist, a Chromium (Cr) layer is spattered as both reference layer for the EBAM machine and as hard mask. About 200 nm of hydrogen silsesquioxane (HSQ) is coated as positive resist for the e-beam lithography. The desired MS pattern appears on the photoresist after development. The lens structure is then transferred into the Cr and SiN_x_ layer by one first step of ion beam etching (IBE) followed by a reactive plasma etching step. Finally, the residual of Cr and photoresist is stripped by wet acid etching.

**
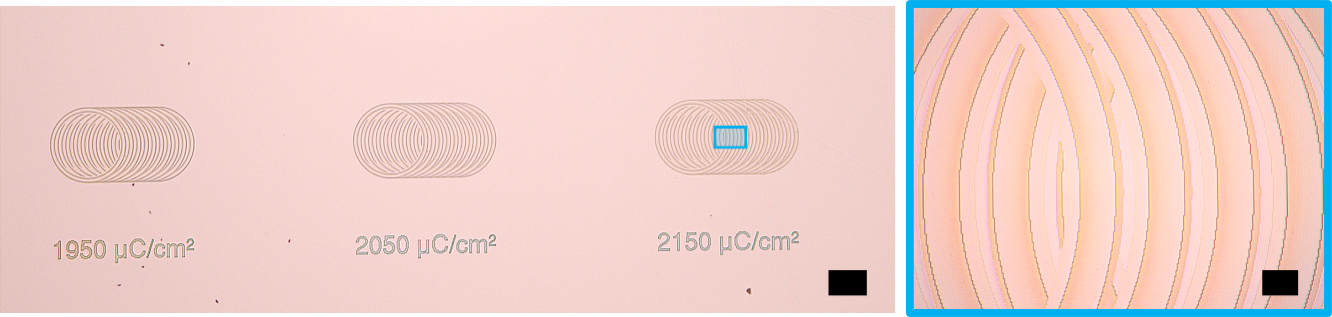
**

**Supplementary Figure 6 – Inspections of the metasurface layouts after e-beam lithography and HSQ development.** Three metasurfaces (MSs) for Bessel beam LLS generation. Focal length 2000 µm. Scale bar: 100 µm (left); 10 µm (right).


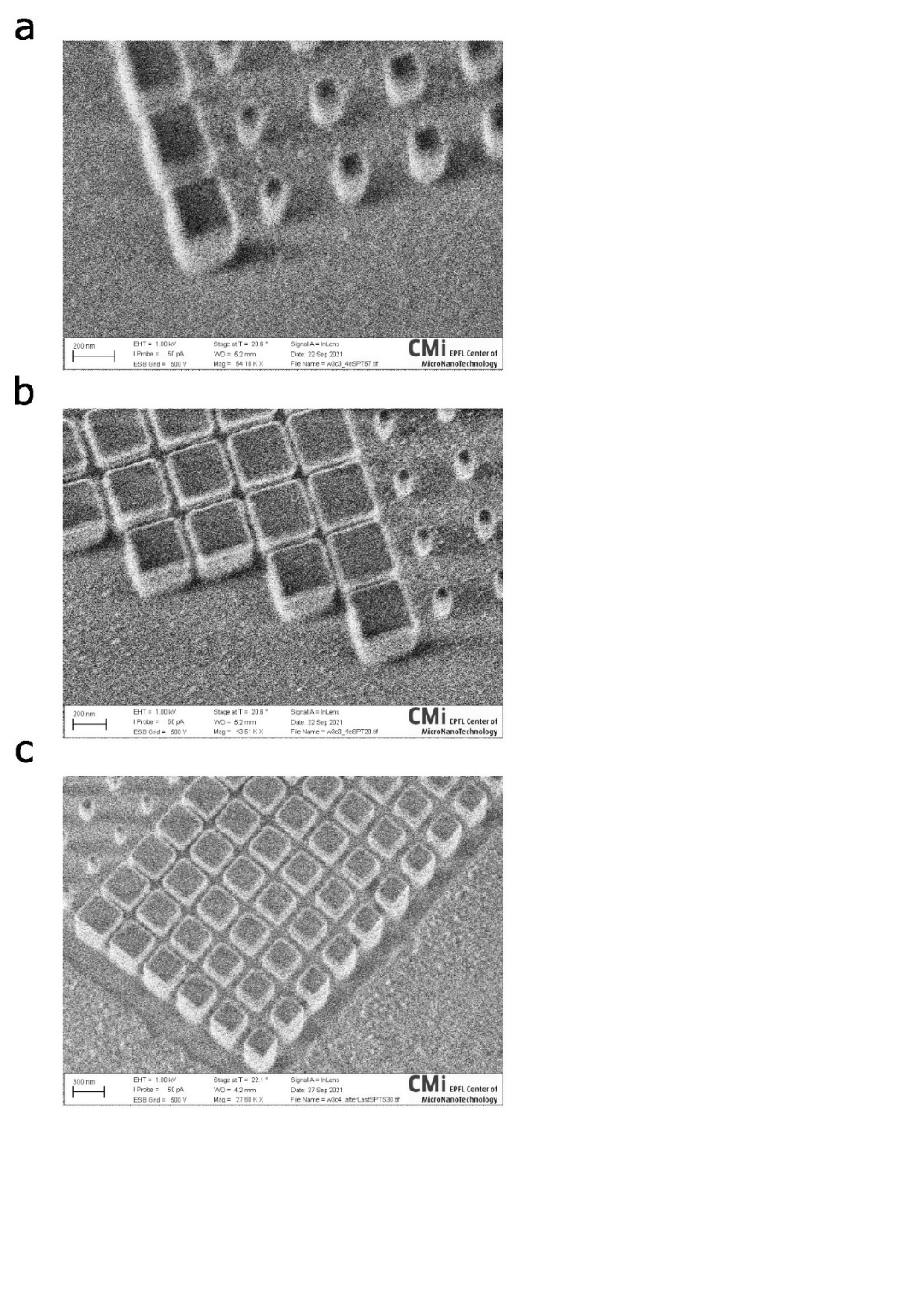


**Supplementary Figure 7 Scanning electron microscopy (SEM) inspections of the metasurface nanopillars.** **a**, and **b**, Scale Bar 200 nm. **c**, Scale Bar 300 nm.

**
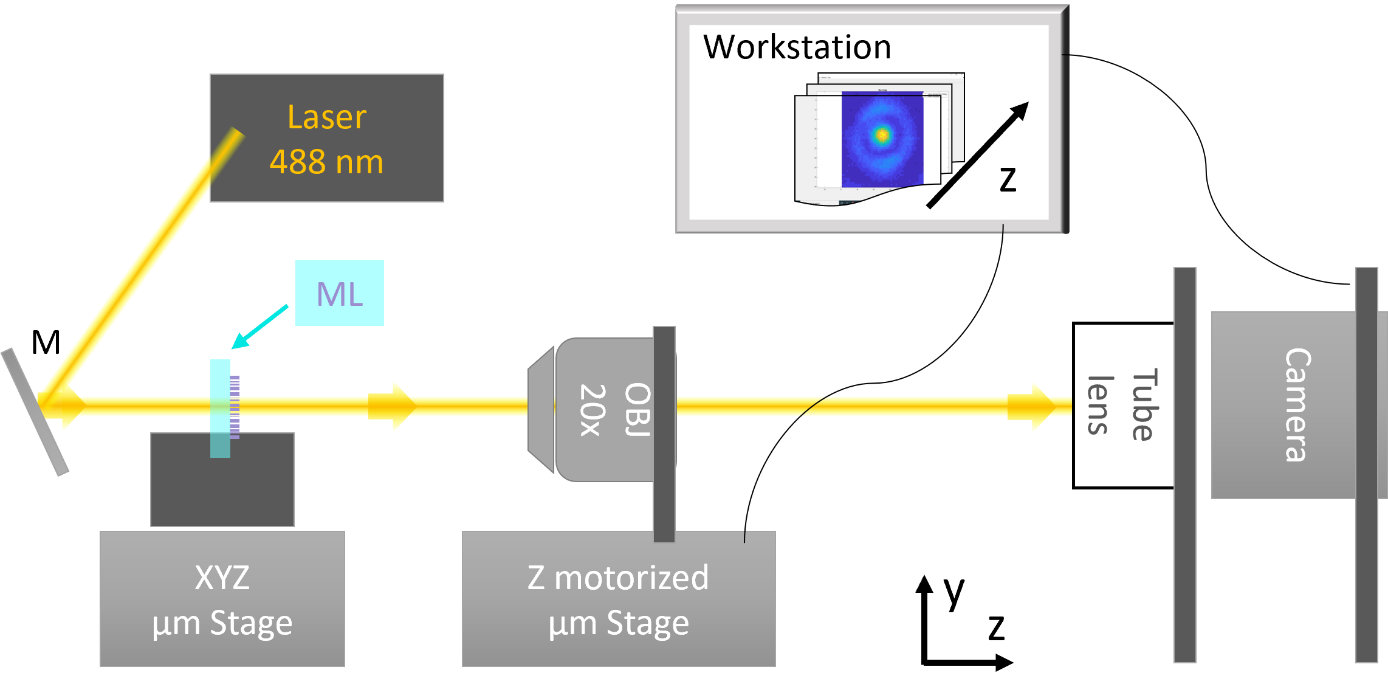
****Supplementary Figure 8 – Sketch of the metasurface characterization setup.** The laser beam is aligned perpendicular to the metasurface (MS) plane. The MS is fixed in the vertical slit of a custom-made holder mounted on a xyz-stage. The detection side is composed by: the 20x Olympus objective (NA = 0.4), the tube-lens (focal length f = 200 mm) and the camera (XIMEA, MQ013RG-ON) connected to the acquisition workstation. The objective is mounted on a motorized stage to automatically acquire the beam profile propagating along the axial (z) direction. Experimental parameters: laser wavelength λ = 488 nm; laser power P = 0.2 mW; camera exposure time Exp = 1 ms; back projected pixel size = 216 nm.


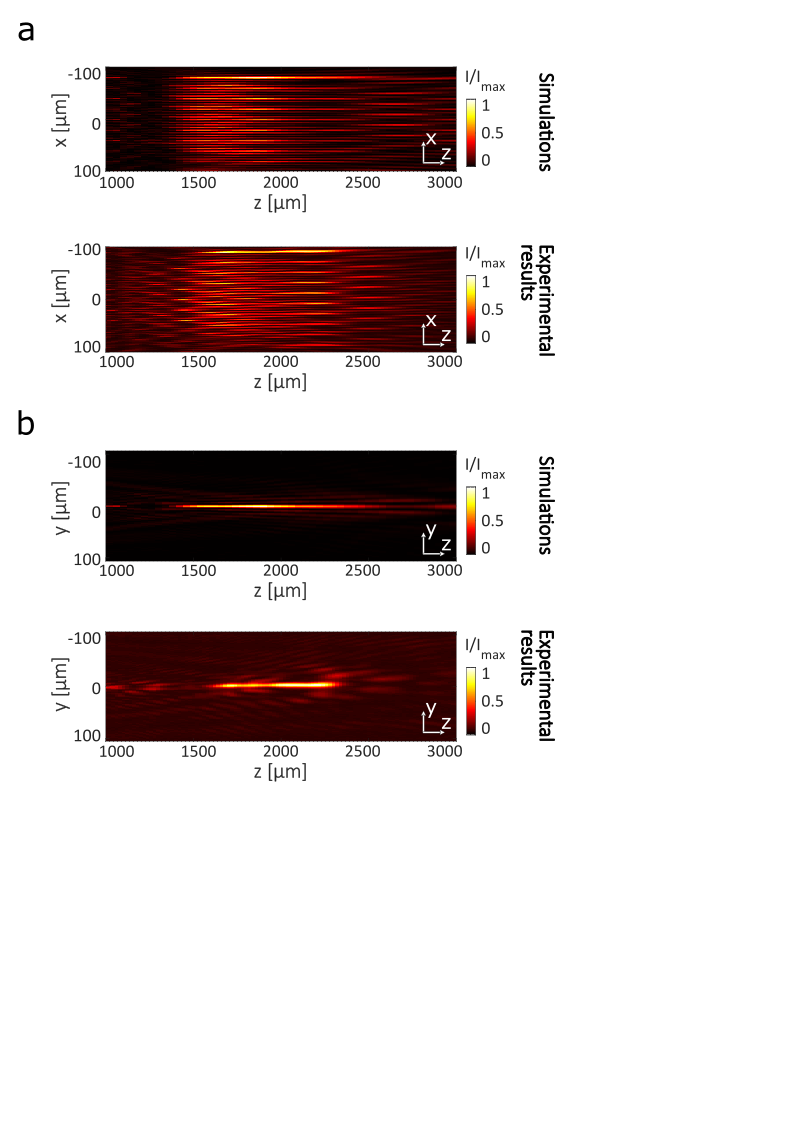


**Supplementary Figure 9 –** **Metasurface based** **Bessel beam lattice light sheet: simulation and characterization.** **a,** Simulation of the XZ beam profile of the BB-LLS generated by our metasurface (MS) design (**top**); experimentally measured beam profiles of the BB-LLS generated by our fabricated MS (**bottom**). **b,** Simulation of the YZ beam profile of the BB-LLS generated by our metasurface (MS) design (**top**); experimentally measured beam profiles of the BB-LLS generated by our fabricated MS (**bottom**). Experimental parameters: laser power P < 0.5 mW; laser wavelength λ = 488nm; camera exposure time Exp = 1 ms.

S5. Achievable uniformity of the MS-based LLS approach in the dithered mode

To compute the light-sheet uniformity achievable with our MS-based LLS method we developed a MATLAB script which simulates the effect of a galvo mirror placed in front of metasurface entrance. The angle between the propagation axis of the input beam and the axis perpendicular to the surface of the MS is increased at each iteration from 0 degree up to the maximum angle required to shift the LLS pattern over a lateral X-range coincident with the LLS period. The larger the LLS period, the larger will be the angular range required to shift the LLS pattern. The MATLAB script performs these following main steps:

1. first the beam incident on the metasurface is tilted at different angles from 0 degree up to the maximum angle required to shift the LLS patter of over a lateral X-range equal to the LLS period;
2. then the beam propagation (the intensity profile at each z-plane) is computed for each of the angular steps and saved in a .tiff stack.

To final dithered mode is computed by summing up the LLS intensity profiles generated with different angles of the input beam at each z-plane.

To cover a lateral X-range of 20 µm, as required for the LLS period generated in this work, we simulated an angular range from 0 degrees to 30 degrees at angular-step of about 1 degree each (35 angular steps). Therefore, to increase the dithering step speed or to decrease the galvo mirror angular range, one can design a LLS with a shorter period. For example, the LLS obtained in this work could have been generated also with a period divided by a factor of two.

As shown in **Supplementary Figure 10** and **Supplementary Figure 11**, the dithered mode can achieve a uniformity higher than 15% over a LS-area of about 200 x 500 ${\mu m}^{2}$ in the XZ plane. The normalized intensity values reported in **Supplementary Figure 13b** are the ratio between the difference of the maximum and minimum intensity values (ΔI) over the average intensity (I_mean_) of the intensity profiles along the X-direction in the XZ plane of **Supplementary Figure 13a**.

**
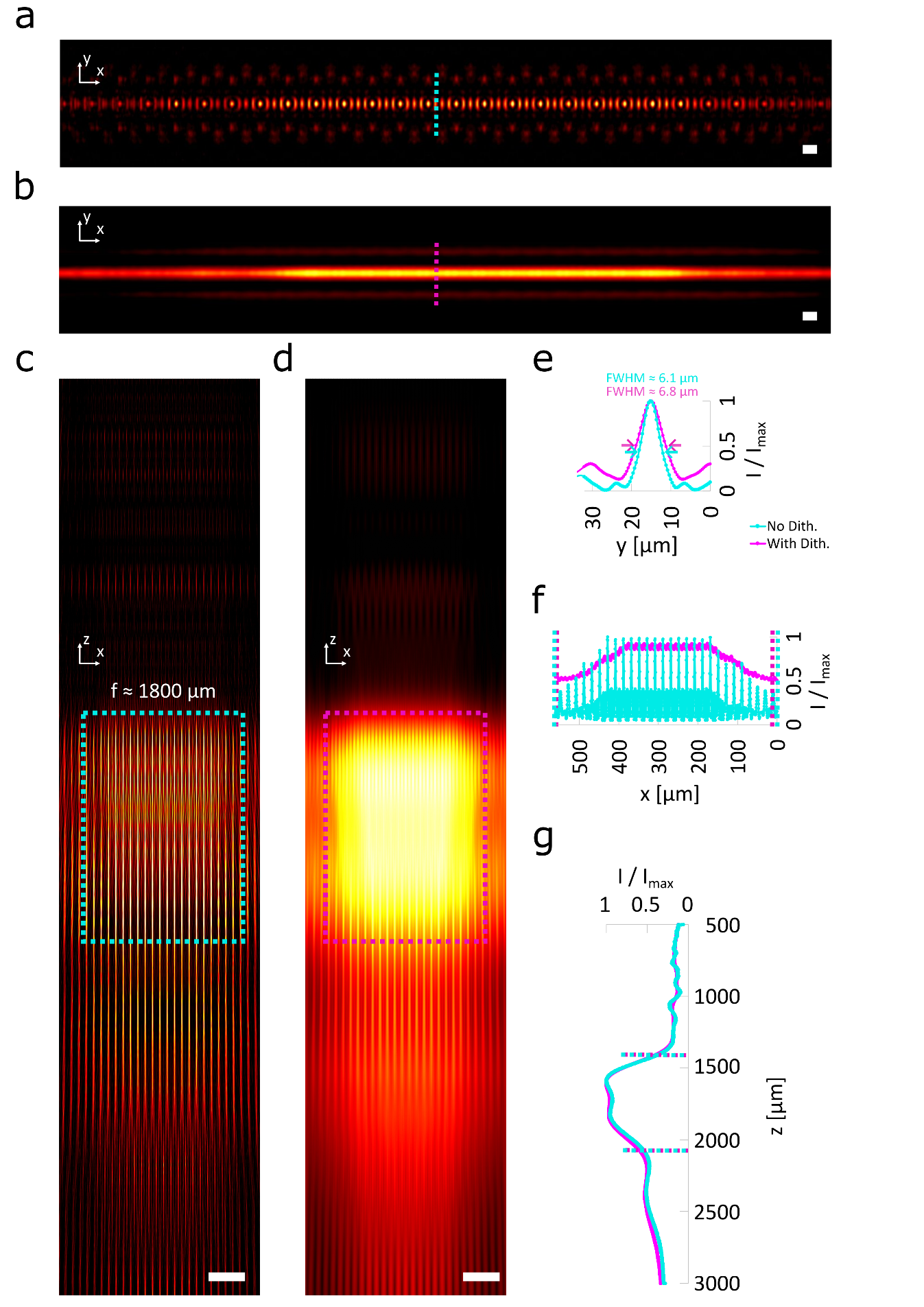
Supplementary Figure 10 –** **MS-based Bessel beam lattice light sheet with and without dithering.** **a,** MS-based LLS profile in the XY plane transversal to the beam propagation without dithering. **b,** MS-based LLS profile in the XY plane transversal to the beam propagation with dithering. **c,** MS-based LLS profile in the XZ plane without dithering. **d,** MS-based LLS profile in the XZ plane with dithering. **e,** LLS 1D profiles along the Y axis of the XY plane shown in **a** (cyan) and in **b** (fuchsia). **f,** LLS 1D profiles along the X axis of the XZ plane shown in **c** (cyan) and in **d** (fuchsia). **g,** LLS 1D profiles along the Z axis of the XZ plane shown in **c** (cyan) and in **d** (fuchsia). Scale bars: 10 µm (**a** and **b**), 100 µm (**c** and **d**).

**
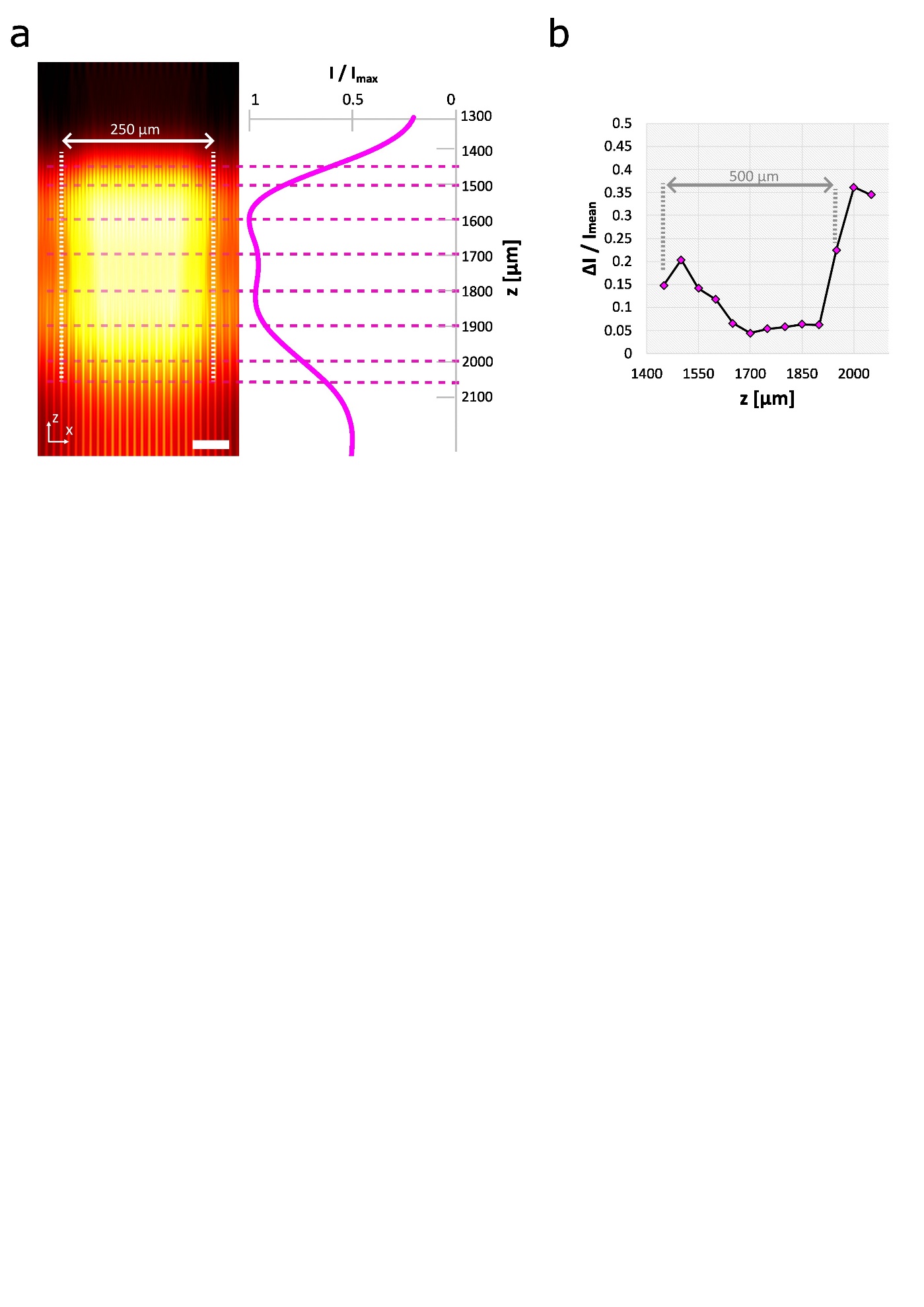
Supplementary Figure 11 –** **MS-based LLS uniformity in dithered mode.** **a,** 1D and 2D intensity profiles of the dithered MS-based LLS in the XZ plane showing the position (fuchsia dashed lines) at which the normalized intensities values are computed. **b,** Normalized intensity values are computed as the ratio between the difference of the maximum and minimum intensity values over the average intensity. The values are measured between z = 1450 µm and z = 2050 µm at step of 50 µm. The plot shows a uniformity of the intensity better than the 15% over a range of about 500 µm. Scale bar 100 µm.

S6. Generation of sub-micrometric MS-based Bessel beam lattice light sheet (BB-LLS)

The method presented in this work can be adapted to obtain metasurfaces capable of generating LLS with arbitrary geometrical parameters: e.g. a LLS with a thinner full-width-at-high-maximum in the Y direction (${FWHM}_{y}$) or a larger full-width-at-high-maximum in the Z direction (${FWHM}_{z}$) than the one proposed above for imaging the zebrafish brain. As proof of concept, we designed other metasurfaces built with the same SiNx nanopillar library presented above that generate different LLS geometries. It is worth noticing that the metasurface can be engineered with the same nanostructures and materials used above such to ensure the use of the same fabrication process flow described above without further development.

As proof of concept, we show: (1) the intensity profile generated by a metasurface encoding a sub-micrometric thick LLS with and without dithering (**Supplementary Figure 12**, **Supplementary Figure 14** and **Supplementary Figure 13**); (2) the intensity profile generated by a metasurface encoding a micrometric thick LLS with an extended size along the X lateral axis (high-aspect ratio: Y-thickness = 1 µm and X-extension = 250 µm – **Supplementary Figure 15** and **Supplementary Figure 16**). Of course, there is always a trade-off between Y-thickness and Z-extension of the beam: as shown in **Supplementary Figure 17**, the thinner the LLS, the shorter is the Z-extension at a fixed delta Δ of the ring (see **Figure 1** for the definition of Δ). From a simple gaussian beam analysis (i.e. assuming a maximum radius R, a Δ of the rings equal to R and an operating wavelength $\lambda=0.488 \mu m$), the numerical aperture NA, the beam waist $w_{o}$ and the Rayleigh length $z_{R}$ are defined by:

| $NA\left( R,f \right)=\sin(\mathrm{atan} \frac{R}{f})$ | ( 7 ) |
| --- | --- |
| $w_{o}\left( R,f \right)=0.42\frac{\lambda}{2NA(R,f)}$; $z_{R}\left( R,f \right)=\frac{\lambda}{\pi{NA}^{2}(R,f)}$ | ( 8 ) |

and therefore the full width at high maximum (FWHM) of the beam in the y and z direction becomes, as described in **Supplementary Figure 17**:

| ${FWHM}_{y}\left( R,f \right)=\frac{4w_{o}\left( R,f \right)}{1.7}$ | ( 9 ) |
| --- | --- |
| ${FWHM}_{z}\left( R,f \right)=2z_{R}\left( R,f \right)$ | ( 10 ) |

Finally, only by decreasing the delta Δ of the rings, the ${FWHM}_{z}$ can be increased without enlarging the thickness ${FWHM}_{y}$ of the LLS (see **Supplementary Figure 18**). However, there is always a compromise between the power transmitted by the ring mask and the beam Z-elongation. Indeed, higher is the thinness of the ring (and thus the beam elongation), higher is the ratio $\varepsilon={{NA}_{min}}/{{NA}_{max}}$ and lower is the transmitted power which is proportional to $T\propto1-\varepsilon^{2}$. In Supplementary Figure 18 we show the simulation of the Bessel beams generated with metasurface with a $R_{in}=R-\Delta$ of the rings of 50 µm, 83 µm and 90 µm respectively. Their resulting transmittance is therefore proportional to $T\propto0.75, 0.31$ and $0.19$ respectively.

**
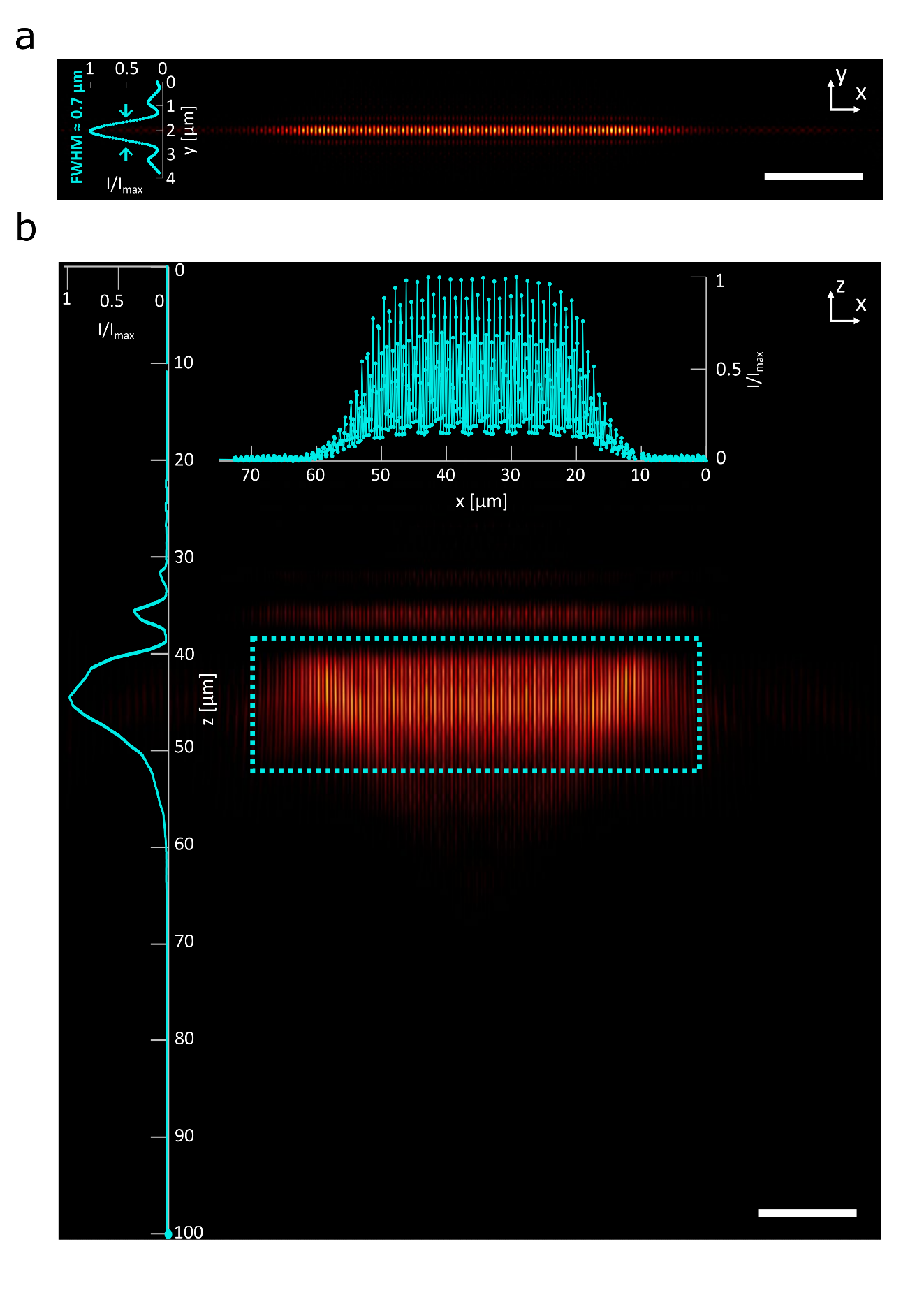
Supplementary Figure 12 – Sub-micrometric** **MS-based Bessel beam lattice light sheet.** **a,** LLS intensity profile in the XY plane. **b,** LLS intensity profile in the XZ plane. Scale bars: 10 µm.

**
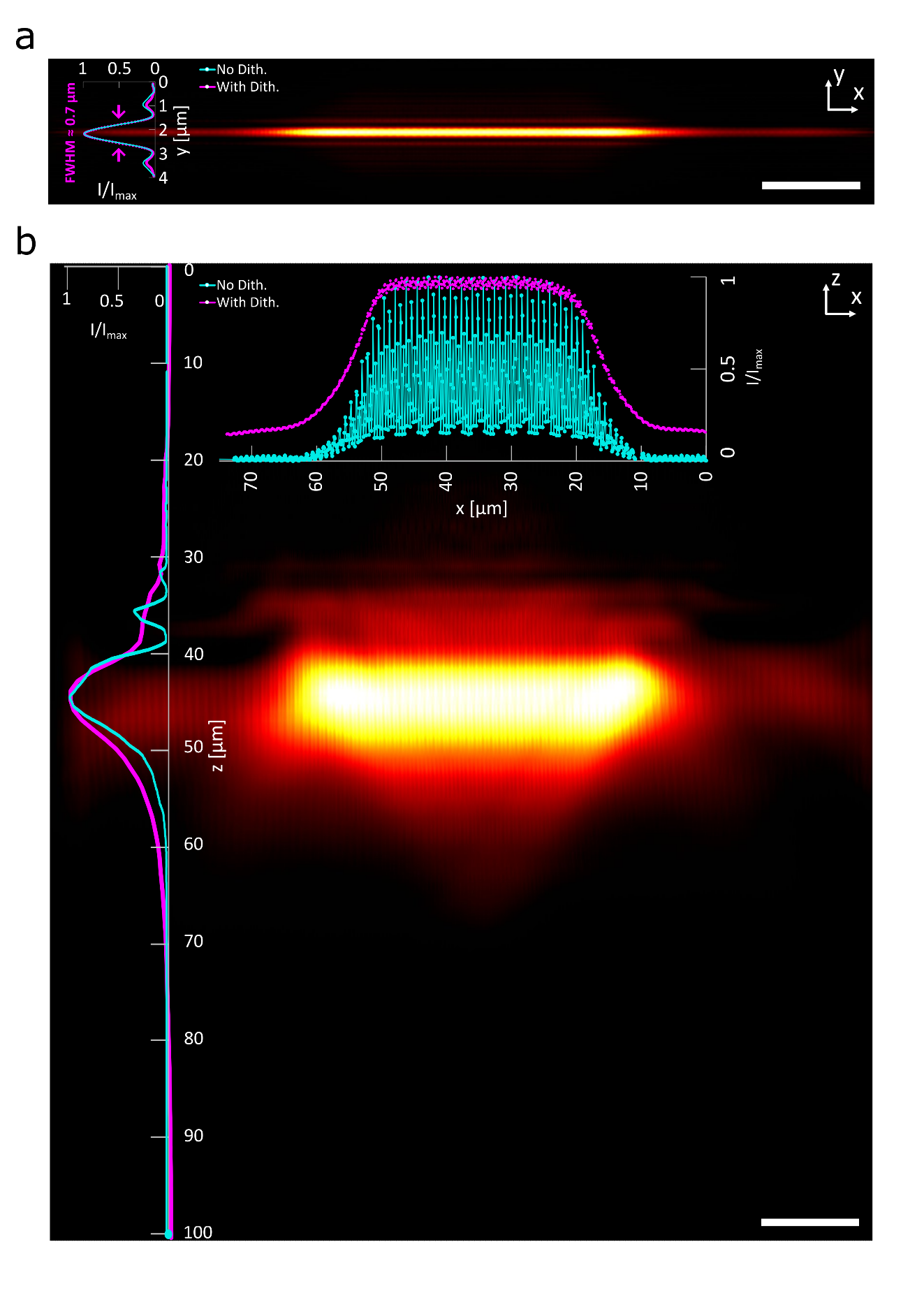
Supplementary Figure 13 –** **Sub-micrometric** **MS-based Bessel beam lattice light sheet in dithered mode.** **a,** LLS intensity profiles with dithering in the XY plane. **b,** LLS intensity profiles with dithering in the XZ plane. Scale bars: 10 µm.

**
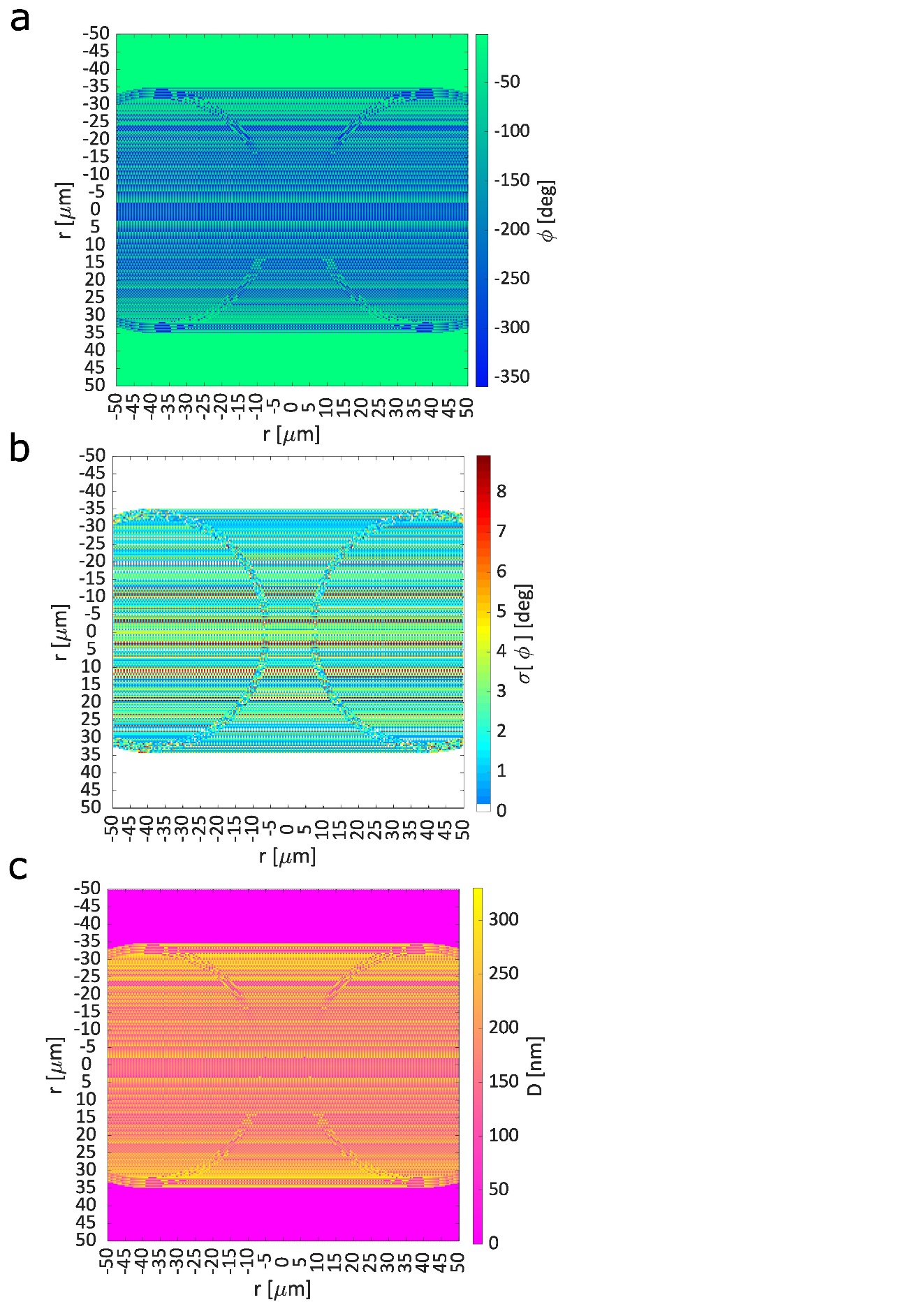
Supplementary Figure 14 – MS-based design for sub-micrometric LLS.** **a,** Phase profile discretized with the library of SiNx nanopillars described in the text above. **b,** Error on the phase: absolute difference between target phase profile values and discretized phase profile values. c**,** Metasurface layout build on the library of SiNx nanopillar described in the text above. Bessel beam period $P = \frac{\lambda}{NA} \sim0.8 \mu m$.

**
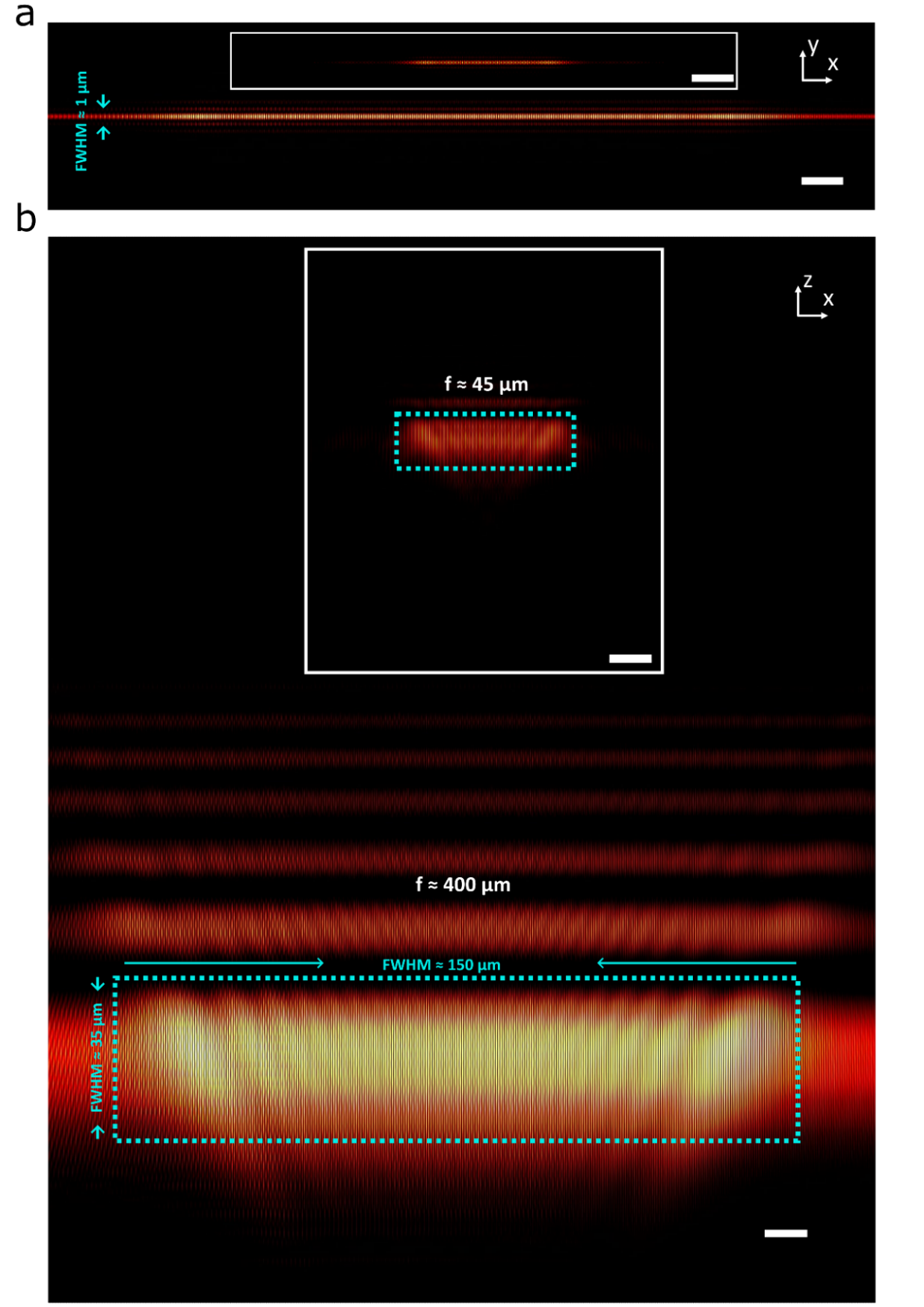
Supplementary Figure 15 –** **XY** **high-aspect ratio MS-based BB-LLS. Generation of a, LLS arbitrarily extended in the lateral X-direction.** **a,** LLS intensity profiles in the XY plane. Inserted dashed box: the sub-micrometric LLS shown in Supp. Fig. 12a. **b,** LLS intensity profiles with dithering in the XZ plane. Inserted dashed box: the sub-micrometric LLS shown in Supp. Fig. 12b. Scale bars: 10 µm.

**
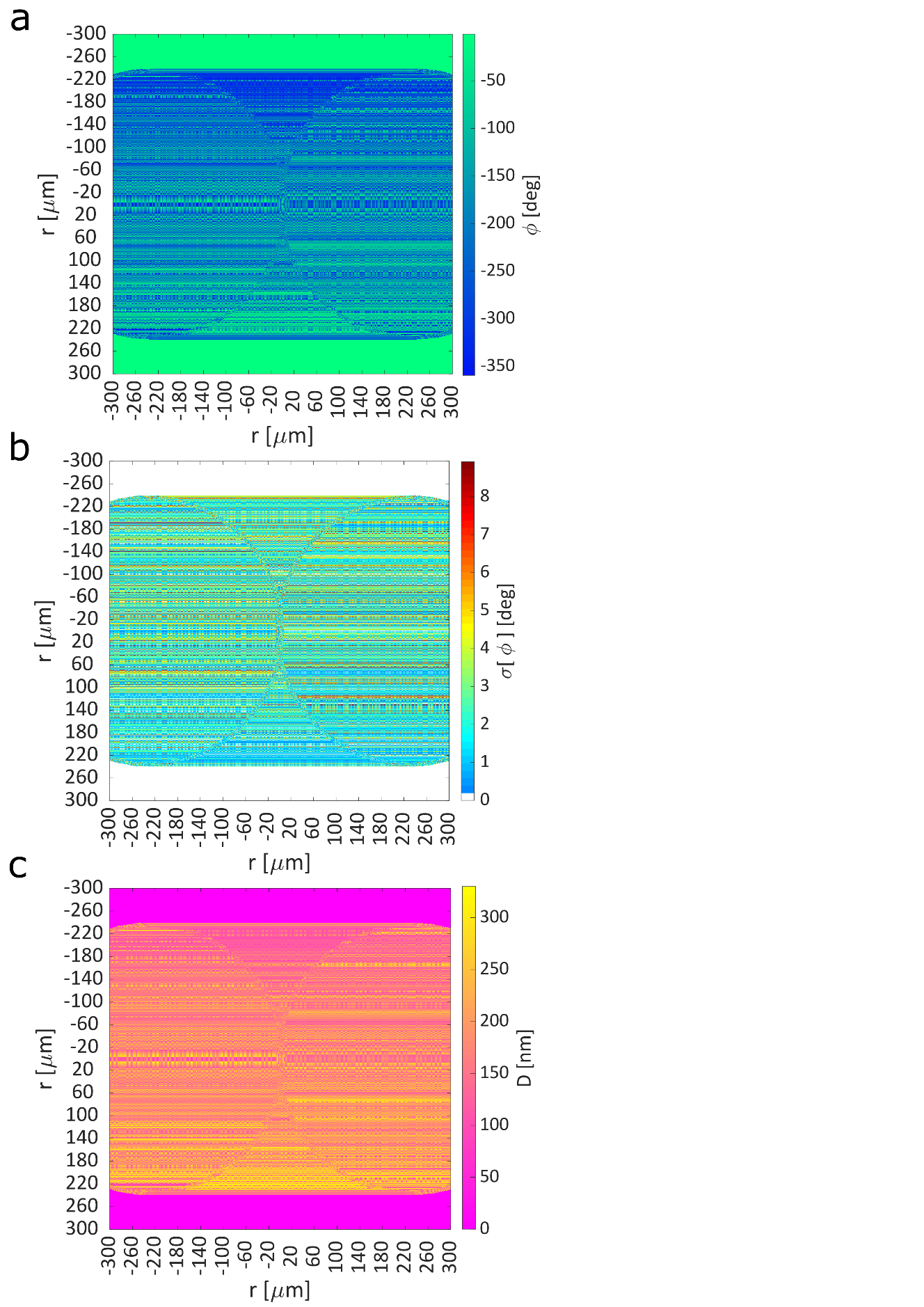
Supplementary Figure 16 – MS-based design for high-aspect ratio micrometric-thick LLS.** **a,** Phase profile discretized with the library of SiNx nanopillars described in the text above. **b,** Error on the phase: absolute difference between target phase profile values and discretized phase profile values. c**,** Metasurface layout build on the library of SiNx nanopillar described in the text above. Bessel beam period $P = \frac{\lambda}{NA} \sim1 \mu m$.

**
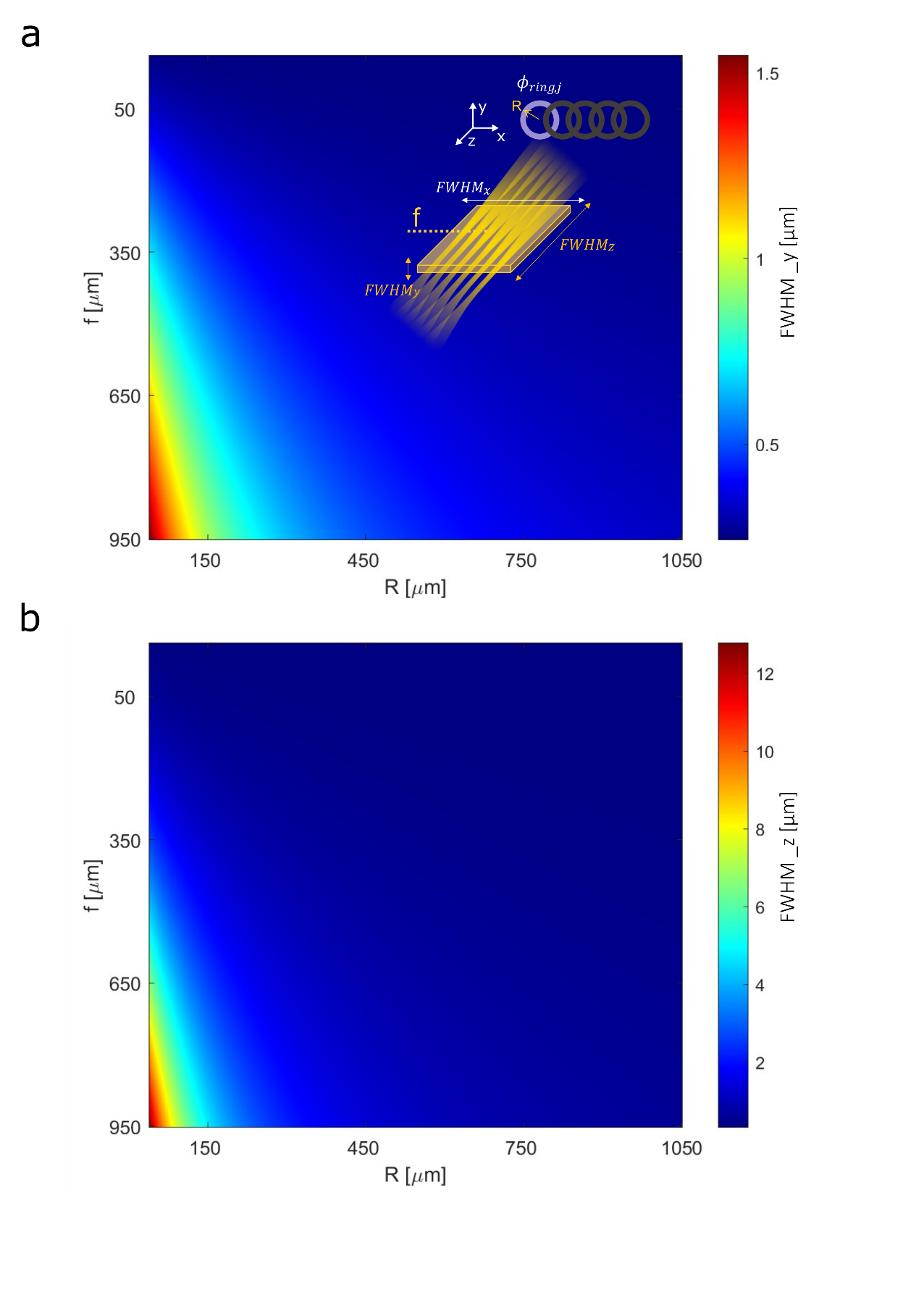
**

**Supplementary Figure 17 –** **Trade-off between FWHM_y_ and FWHM_z_ of the LS.** The thinner the LS, the shorter is the Z-extension at a fixed delta Δ of the rings generating the Bessel beams. **a,** FWHM_y_ as a function of the maximum ring radius R and of the focal length f. **b,** FWHM_z_ as a function of the maximum ring radius R and of the focal length. See Note S6 for the definition of FWHM_y_ and FWHM_z_.

**
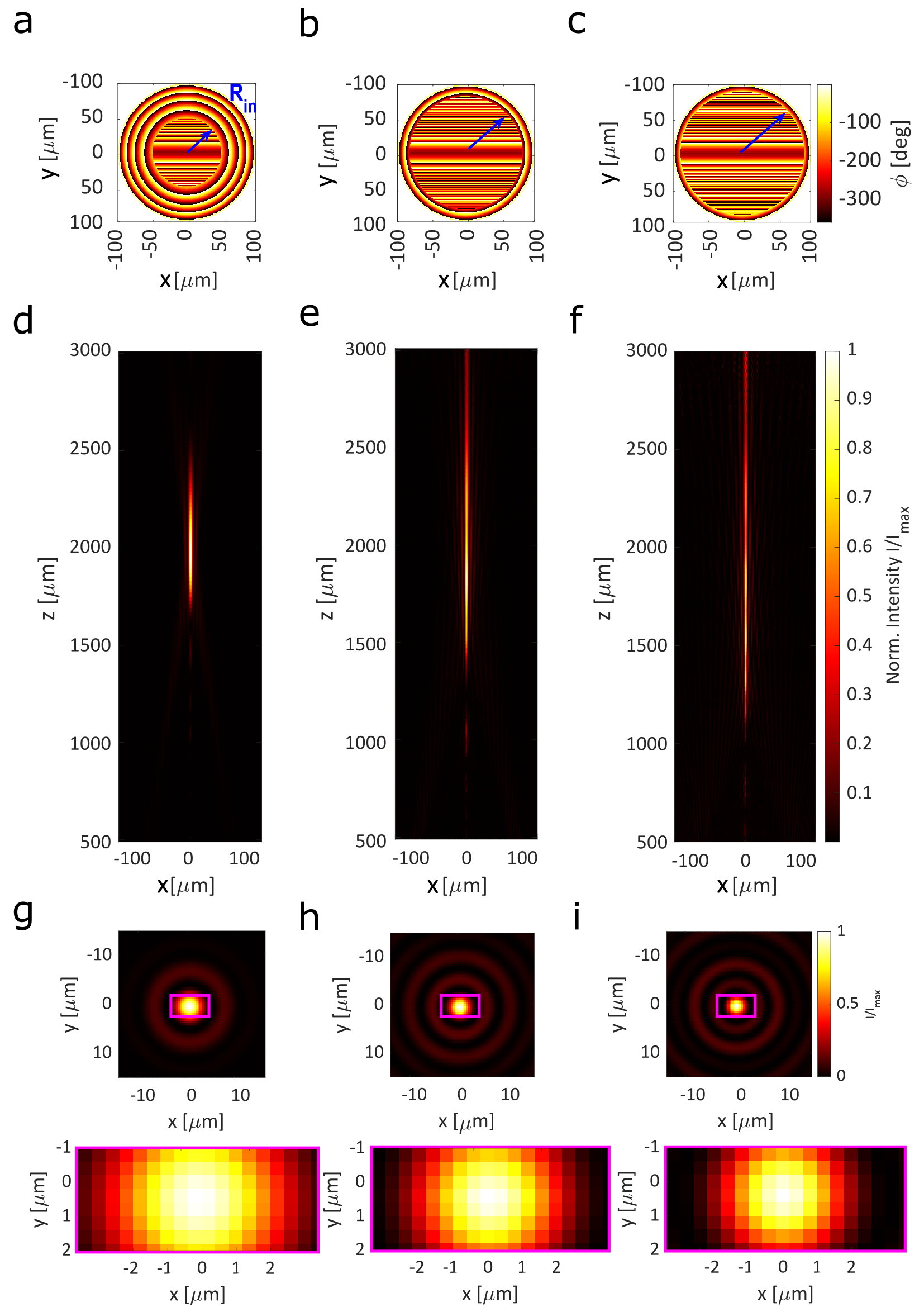
Supplementary Figure 18 –** **Compensation of the FWHM_z_ of the LS by decreasing the ring thickness.** The FWHM_z_ can be extended by decreasing the delta Δ of the rings generating the Bessel beams. **a-c,** Phase profiles of the Bessel beam designed with a $R_{in}$ of 50 µm, 83 µm and 90 µm respectively. Metalens respectively with a focal length of 2000 µm and operating wavelength of 478 nm. **d,** Simulated beam profiles of the Bessel beam generated by the metasurface with $R_{in}$ = 50 µm in the XZ plane. **e,** Simulated beam profiles of the Bessel beam generated by the metasurface with $R_{in}$ = 83 µm in the XZ plane. **f,** Simulated beam profiles of the Bessel beam generated by the metasurface with $R_{in}$ = 90 µm in the XZ plane. **g,** Simulated beam profiles of the Bessel beam generated by the metasurface with $R_{in}$ = 50 µm in the XY plane. The fuchsia box highlights the central area of the XY-PSF (**bottom**). **h,** Simulated beam profiles of the Bessel beam generated by the metasurface with $R_{in}$ = 83 µm in the XY plane. The fuchsia box highlights the central area of the XY-PSF (**bottom**). **i,** Simulated beam profiles of the Bessel beam generated by the metasurface with $R_{in}$ = 90 µm in the XY plane. The fuchsia box highlights the central area of the XY-PSF (**bottom**).

S7. Comparison of MS-based cylindrical light-sheet vs MS-based LLS

We studied the expected performance of our LLS method by comparing, through both simulations and experiments, the intensity profile of our MS-based LLS with the intensity profiles obtained from a cylindrical metalens and from a metasurface generating a single Bessel beam. We designed both the cylindrical metalens and the BB-metasurface with the same input parameter used to generate the LLS and the same SiNx nanopillar library (operating wavelength $\lambda=0.488 \mu m$, outer radius $R=100 \mu m$, focal length $f=2000 \mu m$ and for the single Bessel beam an inner diameter $R_{in}=83 \mu m$).

We measured an LLS thickness larger than the thickness of a single Bessel beam and comparable with the thickness of a LS generated with a simple cylindrical metalens (simulations are shown in **Supplementary Figure 19g-i;** experimental results are shown in **Supplementary Figure 20c**). However, at a given desired light sheet thickness, an LLS has a more than three times larger Z-extension compared to the LS generated by the cylindrical metalens which makes the LLS the best choice form illuminating a large FOV (simulations are shown in **Supplementary Figure 19d-f;** experimental results are shown in **Supplementary Figure 20d**).

It is worth noticing that the X-extension of the light sheet can be extended as much as desired. To prove that the fabrication process is compatible with larger x-extension we fabricated one of the SiNx metasurface with millimetric extension (**Supplementary Figure 20a**).

**
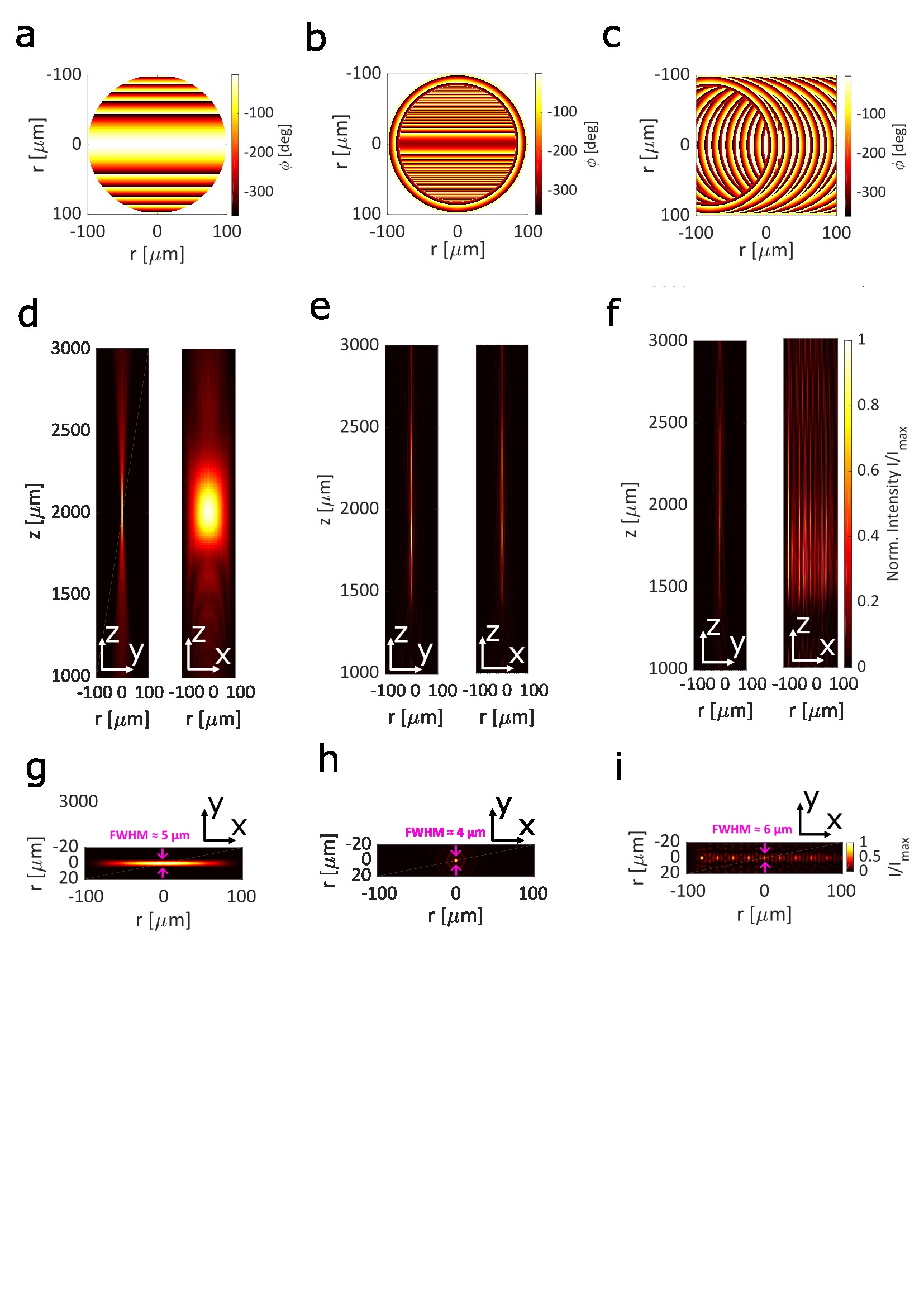
Supplementary Figure 19 – Cylindrical metalens-based LS, MS-based Bessel beam,** **MS-based LLS: design and simulations.** **a,** **b, and c,** Phase profiles of the metasurfaces generating a cylindrical lens LS, a single Bessel beam and a LLS respectively with a focal length of 2000 µm and operating wavelength of 478 nm. **d,** Simulated beam profiles of the LS generated by the cylindrical metalens in the YZ (left) and XZ (right). **e,** Simulated beam profiles of one single Bessel beam generated by the MS in the YZ (left) and XZ (right). **f,** Simulated beam profiles of the Lattice ML on the horizontal YZ (left) and XZ (right) plane. **g,** Simulated beam profile of the LS generated by the cylindrical metalens in the transversal focal plane XY plane. **h,** Simulated beam profile of the single Bessel beam in the transversal focal plane XY plane. **i,** Simulated beam profile of the LLS in the transversal focal plane XY plane.

**
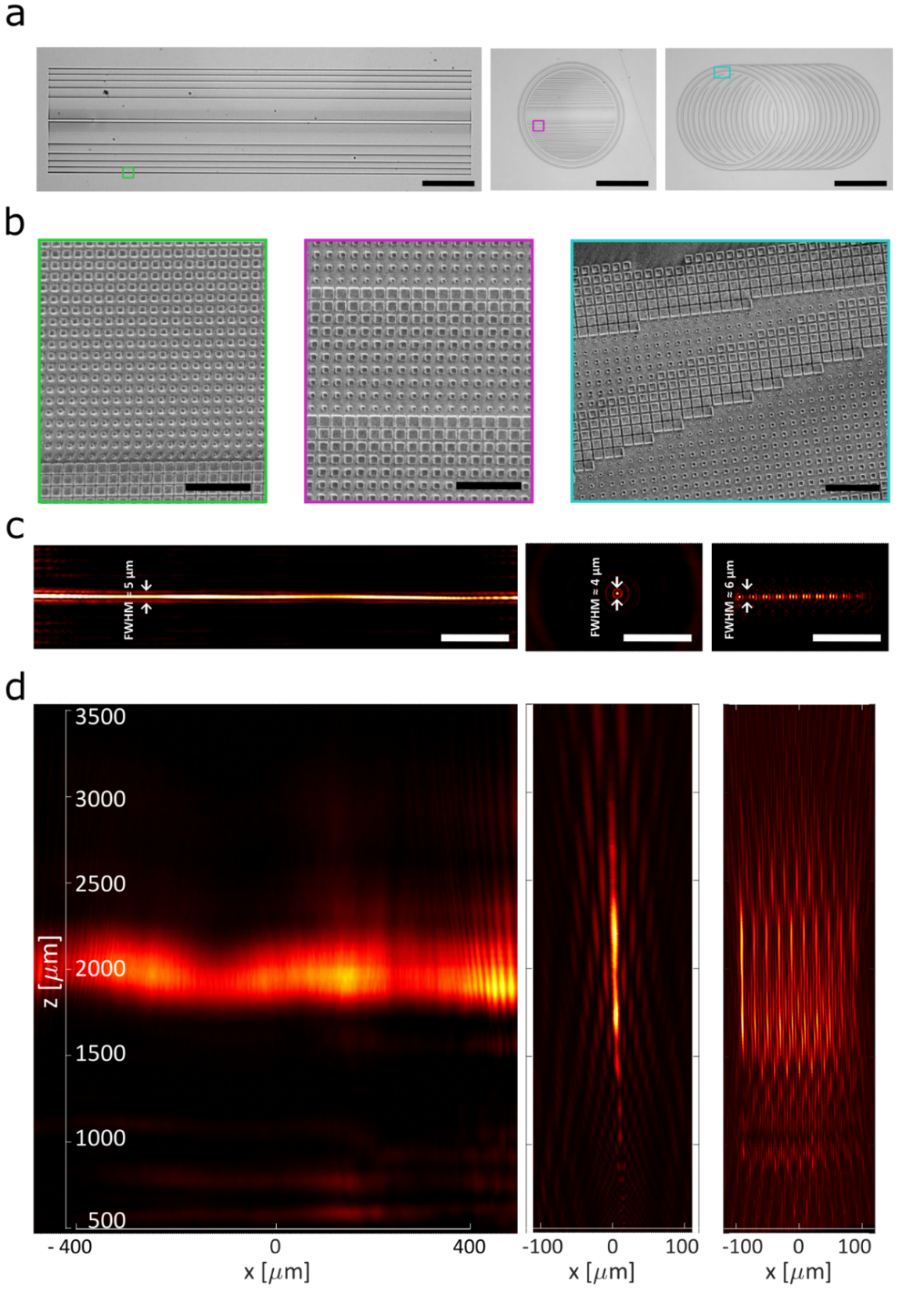
Supplementary Figure 20 –** **Cylindrical metalens-based LS, MS-based Bessel beam,** **MS-based LLS: experimental results. a,** Optical wide field images of the metasurface generating the LS with a cylindrical lens phase profile (left), the single Bessel beam (center) and the lattice light sheet (LLS). **b,** Scanning electron microscopy (SEM) images of the parts of the metasurfaces highlighted with colored dashed boxes in **a**. The SEM images show the nanometric scaling of the square nano-scatter cross-section. **c,** Measured XY intensity profiles of the beam propagating from the cylindrical lens profile (left), from the metasurface generating a single Bessel beam (center) and from the metasurface generating a lattice light sheet (LLS). **d,** Measured XZ intensity profiles of the beam propagating from the cylindrical lens profile (left), from the metasurface generating a single Bessel beam (center) and from the metasurface generating a lattice light sheet (LLS). Scale bars: **a,** 100 µm; **b,** 2 µm; **c,** 100 µm.


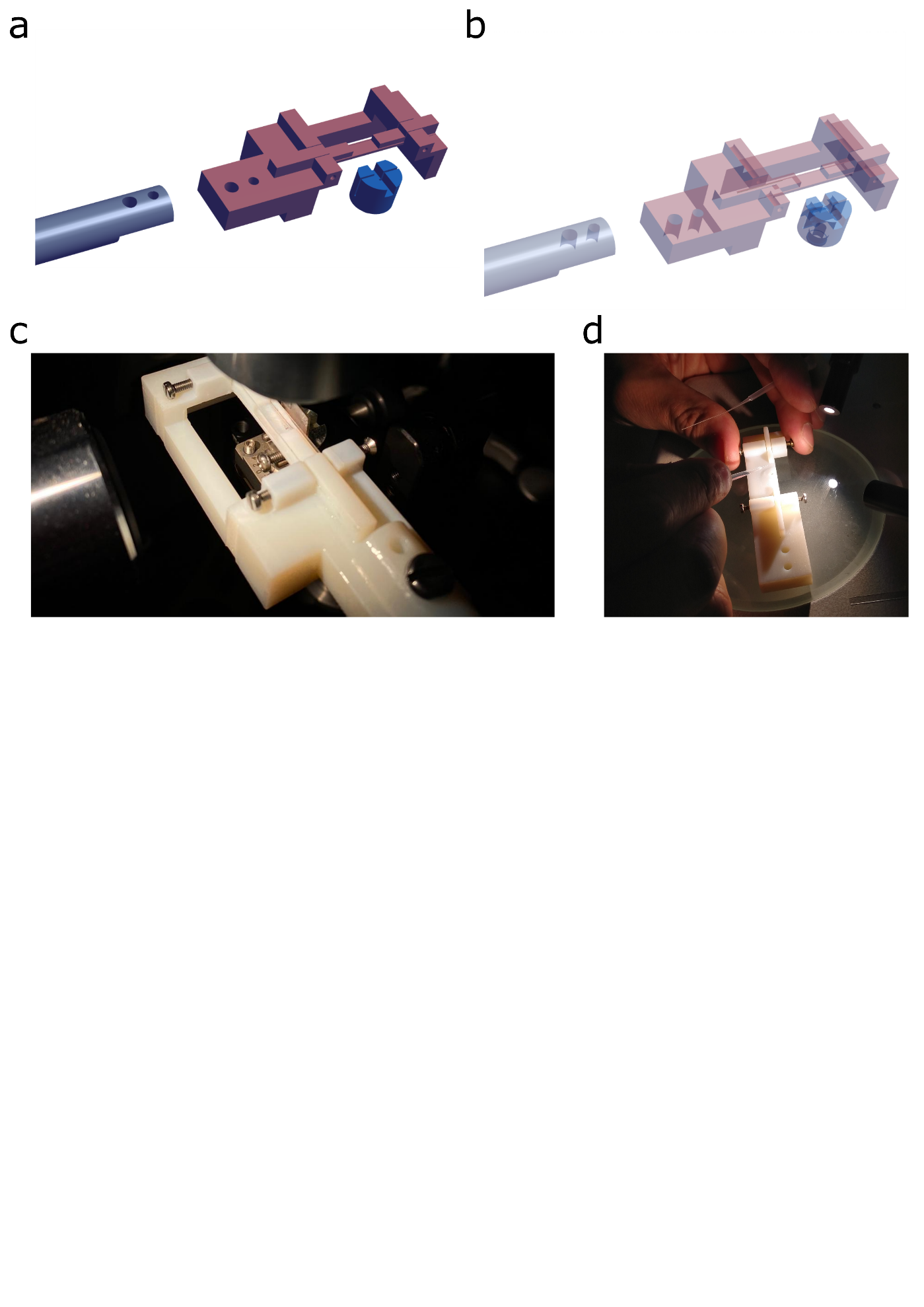


**Supplementary Figure 21 –** **Custom made holder designs for MS-chip mounting and zebrafish imaging.** **a,** 3D solid views of the metasurface chip holder (dark-blue) and the 3D-printed holder for zebrafish embedding (brown). **b,** 3D transparent views of **a**. **c,** 3D-printed zebrafish holder with the zebrafish embedded in agarose within the imaging well. The edges of the zebrafish well are 170 um thick glass slices cut with a rectangular shape (3 mm x 40 mm) and inserted in the corresponding slits engraved in the 3D-printed holder. **d,** One of the steps of the zebrafish embedding process: the sagittal plane of the zebrafish is manually oriented parallel to the metasurface chip immediately after placing the zebrafish larva within the agarose drop and before the agarose hardens.


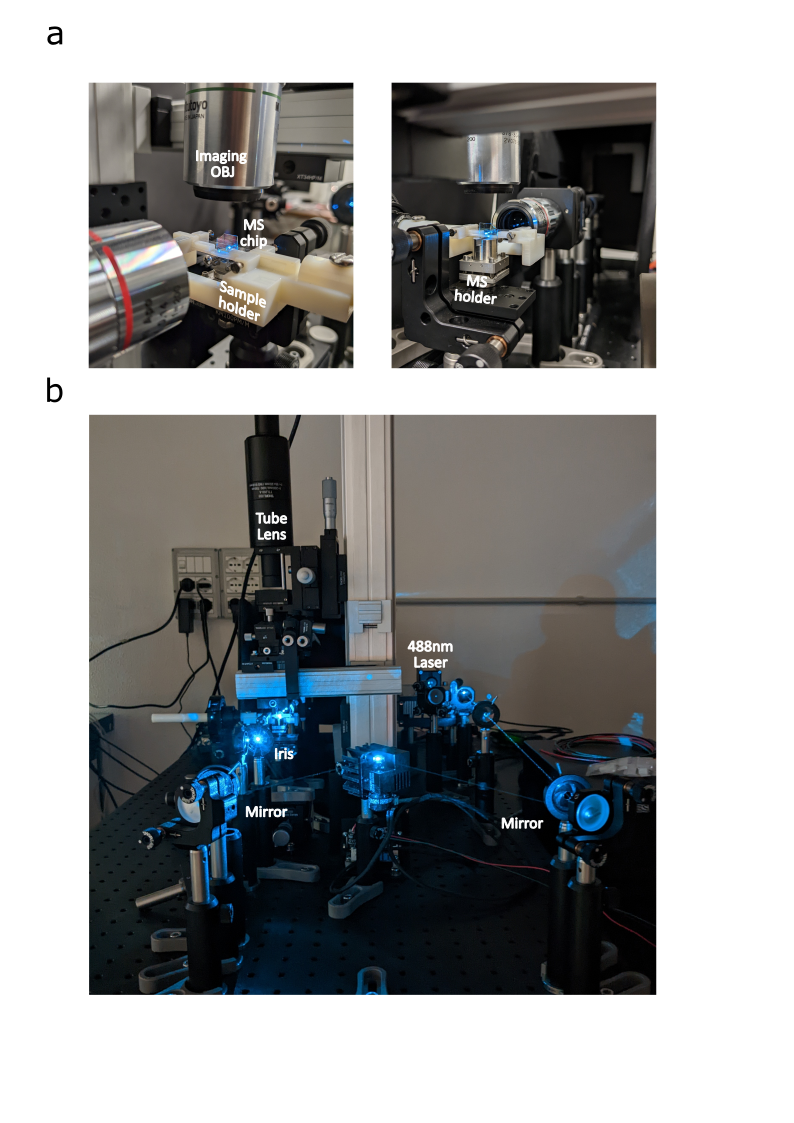


**Supplementary Figure 22 –** **MS-based Bessel beam lattice light sheet microscope.** Photos of our custom metasurface holder and zebrafish holder (**top**) and of our MS-based BB-LLS setup (**bottom**).


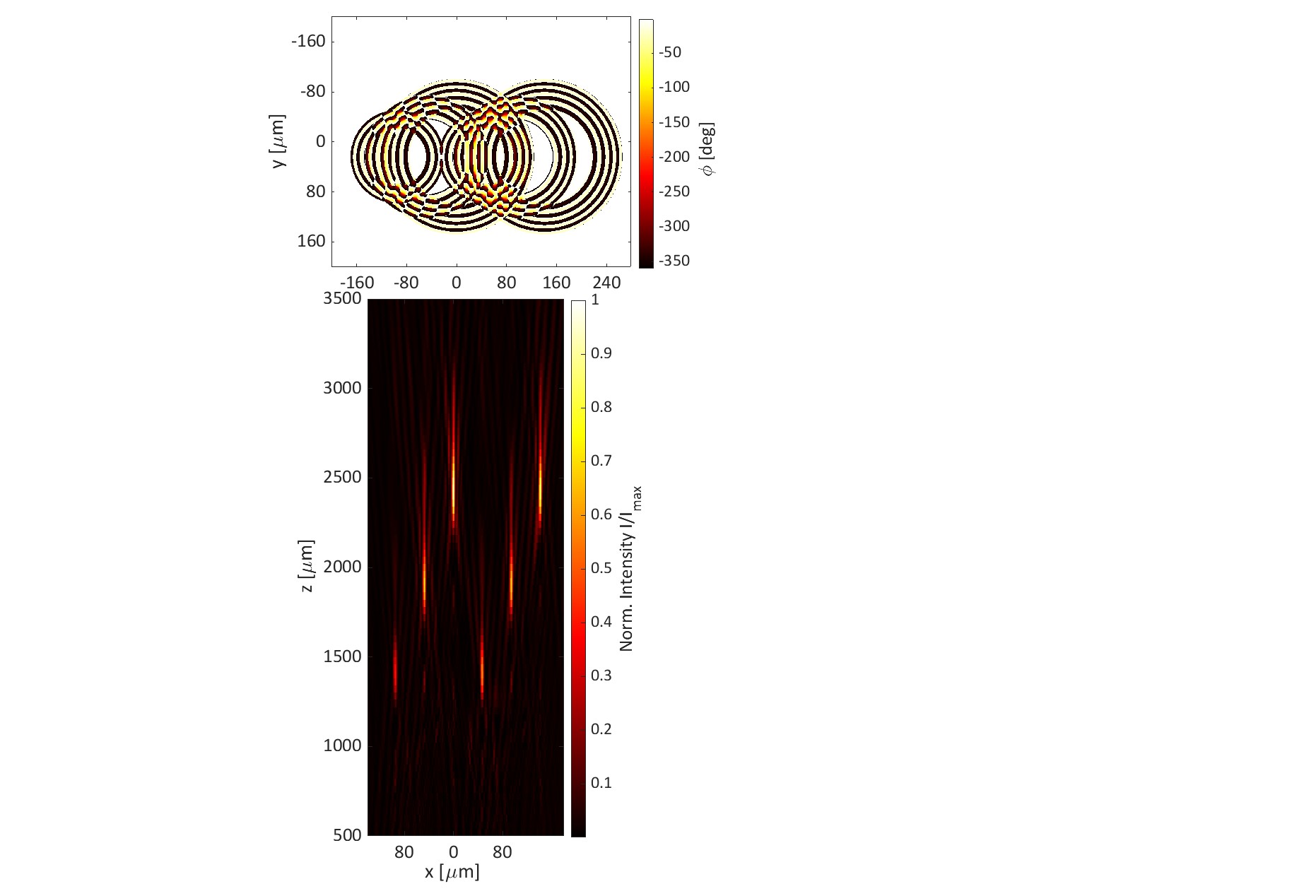


**Supplementary Figure 23 –** **Example of a simulated** **MS-based lattice of Bessel beams with different focal length.** Phase profile (**top**) and resulting propagation beam profile (**bottom**). In this example, the six Bessel beams have been generated with three different focal lengths, 1500 µm, 2000 µm and 2500 µm. Operating wavelength of 478 nm.

**
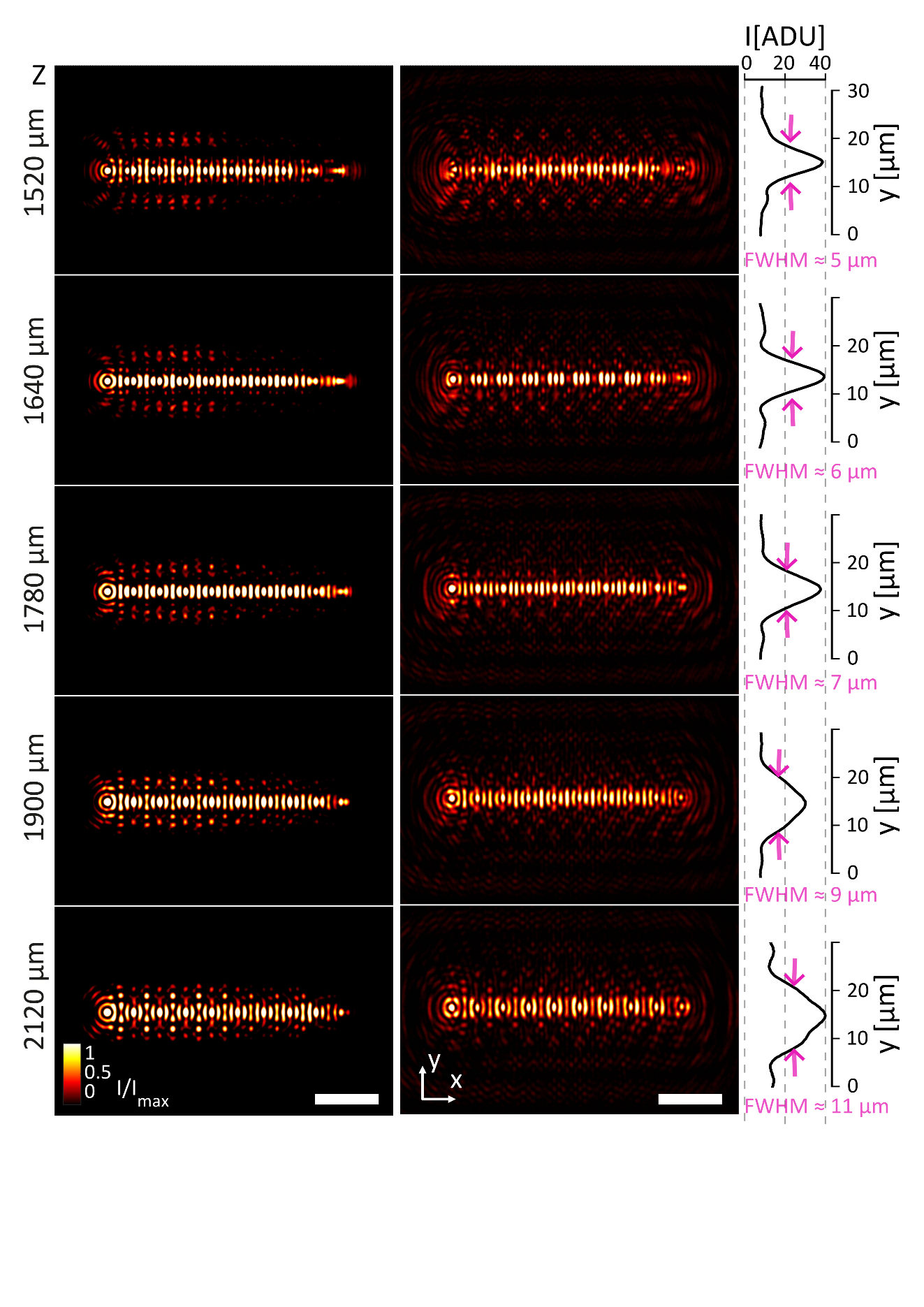
**

**Supplementary Figure 24 –** Cross section of the Bessel beam lattice light sheet at various propagation depths. Simulated results versus experimentally measured results. Operating wavelength: $\lambda=478 nm$. Input laser power 1mW. The BB light sheet profiles were acquired at different propagation positions z, from z0 = 500 µm up to z = 3500 µm with a step size of z_step_ = 5 µm with an exposure time of 1 ms and laser power value of P_w_ ~ 0.3 mW. The BB-LLS thickness was then estimated by measuring the full width at half maximum (FWHM) of the X and Y intensity profile at a depth ranging from 1500 µm up to 2200 µm every 120 µm.

Supplementary Tables

Fabrication process

| Step | Process description |
| --- | --- |
| 01 | SiNx low-stress LPCVD deposition  (520 nm - metasurface layer) |
| 02 | Cr sputtering  (40 nm) |
| 03 | HSQ coating (1700 rpm, 180nm) |
| 04 | E-beam lithography  (Dose: 1950:2150 uC/cm^2^, 1 nA, 33MHz, @100 kV) |
| 05 | HSQ development  (3 min) |
| 06 | Cr etching (mask) (30 sec) |
| 07 | SiNx plasma etching (metasurface realization)  (SPTS APS, CHF_3_/SF_6_ chemistry; etch time: 12 min) |
| 08 | Cr wet etching (Cr_01_UN3264, 3 min) |

**Supplementary Table 1** Chip fabrication process: main steps

Experimental condition and localization results

| Sample/Figure | **Exposure time**  **[ms]** | **Laser λ [nm]** | **Laser nominal [mw]** | **Laser at the sample [mW]** | **Number of Frames** | **Raw Image pixel size [nm]** | **Analysis Algorithm** |
| --- | --- | --- | --- | --- | --- | --- | --- |
| Fig.2 | 1 | 488 | 22mA/0.5mW | - | 200 | 216 | Custom MATLAB routine |
| Fig.3a | 500 | 488 | 60 | 1 | 400 | 240 | Suite2P[5] |
| Fig.3b |  |  |  |  |  |  |  |
| Fig.3c |  |  |  |  |  |  |  |
| Fig.3d |  |  |  |  |  |  |  |

**Supplementary Table 2** Experimental condition and dataset analysis information.

Supplementary References

[1] U. S. Kamilov *et al.*, “Optical Tomographic Image Reconstruction Based on Beam Propagation and Sparse Regularization,” *IEEE Trans. Comput. Imaging*, vol. 2, no. 1, pp. 59–70, Mar. 2016, doi: 10.1109/TCI.2016.2519261.

[2] M. D. Feit and J. A. Fleck, “Beam nonparaxiality, filament formation, and beam breakup in the self-focusing of optical beams,” *JOSA B*, vol. 5, no. 3, pp. 633–640, Mar. 1988, doi: 10.1364/JOSAB.5.000633.

[3] A. Archetti, R.-J. Lin, T. V. Tsoulos, F. Kiani, N. Restori, and G. Tagliabue, “Thermally reconfigurable varifocal silicon metalens,” in *Current Developments in Lens Design and Optical Engineering XXII*, SPIE, Aug. 2021, pp. 42–47. doi: 10.1117/12.2599593.

[4] A. Edelstein, N. Amodaj, K. Hoover, R. Vale, and N. Stuurman, “Computer Control of Microscopes Using µManager,” *Curr. Protoc. Mol. Biol.*, vol. 92, no. 1, p. 14.20.1-14.20.17, 2010, doi: 10.1002/0471142727.mb1420s92.

[5] “Suite2p: beyond 10,000 neurons with standard two-photon microscopy | bioRxiv.” Accessed: Nov. 15, 2022. [Online]. Available: https://www.biorxiv.org/content/10.1101/061507v2
